# Metal oxide nanoparticles embedded in porous carbon for sulfur absorption under hydrothermal conditions

**DOI:** 10.1038/s41598-023-36395-8

**Published:** 2023-06-20

**Authors:** Hang Xiang, David Baudouin, Frédéric Vogel

**Affiliations:** 1 Laboratory for Bioenergy and Catalysis, Paul Scherrer Institute (PSI), 5232 Villigen PSI, Switzerland; 2 University of Applied Sciences Northwestern Switzerland (FHNW), 5210 Windisch, Switzerland

**Keywords:** Chemistry, Energy science and technology, Materials science

## Abstract

MO_x_ (M = Zn, Cu, Mn, Fe, Ce) nanoparticles (NPs) embedded in porous C with uniform diameter and dispersion were synthesized, with potential application as S-absorbents to protect catalysts from S-poisoning in catalytic hydrothermal gasification (cHTG) of biomass. S-absorption performance of MO_x_/C was evaluated by reacting the materials with diethyl disulfide at HTG conditions (450 °C, 30 MPa, 15 min). Their S-absorption capacity followed the order CuO_x_/C > CeO_x_/C ≈ ZnO/C > MnO_x_/C > FeO_x_/C. S was absorbed in the first four through the formation of Cu_1.8_S, Ce_2_S_3_, ZnS, and MnS, respectively, with a capacity of 0.17, 0.12, 0.11, and 0.09 mol_S_ mol_M_^−1^. The structure of MO_x_/C (M = Zn, Cu, Mn) evolved significantly during S-absorption reaction, with the formation of larger agglomerates and separation of MO_x_ particles from porous C. The formation of ZnS NPs and their aggregation in place of hexagonal ZnO crystals indicate a dissolution/precipitation mechanism. Note that aggregated ZnS NPs barely sinter under these conditions. Cu(0) showed a preferential sulfidation over Cu_2_O, the sulfidation of the latter seemingly following the same mechanism as for ZnO. In contrast, FeO_x_/C and CeO_x_/C showed remarkable structural stability with their NPs well-dispersed within the C matrix after reaction. MO_x_ dissolution in water (from liquid to supercritical state) was modeled and a correlation between solubility and particle growth was found, comforting the hypothesis of the importance of an Ostwald ripening mechanism. CeO_x_/C with high structural stability and promising S-absorption capacity was suggested as a promising bulk absorbent for sulfides in cHTG of biomass.

## Introduction

Biomass, such as wood, algae, manure, industrial and domestic residual biogenic waste, etc., is a ubiquitous and renewable source of fuel with a low inherent carbon footprint^1^. Various processes have been developed to produce refined fuels from biomass. Hydrothermal gasification (HTG) is of particular interest to convert wet biomass to gaseous fuels (biogas) applying supercritical water (SCW, T > 374 °C, P > 22.1 MPa)^2^. It features a high thermal efficiency (70–77%) for wet biomass (e.g. sewage sludge) and short residence time of few minutes^3,4^.

For the production of methane-rich biogas, ruthenium (Ru) is known as the most effective active phase under the harsh SCW conditions used^5–7^. DFT calculations indicated that stepped Ru surfaces act as “bond scissors” for adsorbed carbonaceous molecules, breaking down molecules into atomic adsorbates^8^. The most stable adsorbates are atomic carbon and hydrogen, which can then recombine with adsorbed oxygen to form H_2_, CO_2_, CO, CH_4_ in equilibrium with the surrounding media. This feature allows ruthenium to reach thermodynamic equilibrium faster than any other metal^9^, which easily allows a CH_4_ content in the produced biogas over 50 vol.%^10,11^.

However, Ru inherently shows poor tolerance to sulfur, particularly at an oxidation state of 0 to -II. Waldner et al. showed that as low as 16 ppm of S in the feed is sufficient to poison a Ru/C catalyst during continuous gasification of ethanol and synthetic liquefied wood^12^. Dreher et al. firstly studied the poisoning mechanism of Ru/C used for the reforming of biomass in SCW by operando EXAFS combining with isotope labeling. They found a partial surface coverage (about 40%) of S on Ru/C to be sufficient to block the active sites of the catalyst, and indicated the irreversible properties of S-poisoning: more specifically, the catalytic activity of S-poisoned Ru/C could not be restored by flushing the catalyst with sulfur-free feed or pure SCW^7^.

Elliott et al. firstly introduced the application of an affordable sulfur scrubbing bed installed upstream of the Ru/C catalyst for continuous cHTG at 350 °C and 20 MPa, with different microalgae species as the feeds^13^. Raney nickel was used as an S-absorption material, which, however, presented insufficient stream desulfurization abilities. Nickel is also generally known to have low stability under reductive supercritical water conditions and suffers from sintering and leaching^9^. Peng et al. used a commercial ZnO-based S-absorbent to protect the Ru/C (5% Ru) catalyst during a continuous cHTG campaign with Chlorella Vulgaris^14^. The S-rich microalgae feed was successfully gasified to a methane-rich gas during the first 60 h time on stream, reflecting the good performance of the S-absorption by the ZnO material. But a significant accumulation of Zn-based debris was observed on the downstream Ru/C catalyst^15^, which was assumed to be a contributory cause of the catalyst deactivation after 60 h. The instability of the ZnO’s binder (alumina) was proved by XRD, which underwent drastic phase transformations after SCW exposure, resulting in significant decrease of the specific surface area and pore volume of this commercial S-absorbent material. A stable binder material is desired which would preserve the integrity of the S-absorbent grain and prevent the loss of the S-absorbing phase (metal oxide) from streaming down to the catalyst bed.

In supercritical water, only a few oxides show long-term stability. Indeed, mostly refractory oxides (α-Al_2_O_3_, monoclinic ZrO_2_, rutile TiO_2_) proved to withstand such harsh conditions, where other phases or other oxides showed phase transformation, porosity collapse, or dissolution^9,10,16^. Aside from oxides, carbon is one of the few materials that show long-term stability of its porous framework and particle integrity under SCW^9,17,18^, making it a promising low-cost binder material for such conditions. Apart from ZnO, the oxides of molybdenum (Mo), manganese (Mn), and cerium (Ce) also showed good sulfur absorption capacity with intermediate activity in decreasing organosulfur concentration, and Cu(0) demonstrated outstanding performance for both desulfurization and S-absorption^2,19–21^. It is therefore expected that a composite of porous carbon and high loading of metal (or metal oxides) will be an ideal S-absorbent, showing both high S-absorption capacity and structural stability under SCW.

For the synthesis of carbon-supported metal oxide materials, a variety of methods have been developed, including physical mixing and wet chemical processes. Sharma et al. developed a simple ion-exchange method for preparing porous NiO/C catalysts for cHTG. This catalyst showed high activity and sintering resistance even after 50 h of reaction time, compared to other NiO/C catalysts reported in the literature^20^.

In this publication, we developed the synthesis of MO_x_/C (M = Zn, Cu, Fe, Mn, and Ce) sulfur-absorbing materials to achieve a high loading of the active phase MO_x_ (high absorption capacity) nanoparticles (NPs) and a porous C framework. Batch tests were then performed to assess their stability in SCW and S-absorption capacity using an organic S-rich aqueous solution under cHTG conditions. The evolution of the materials upon the two treatments was carefully assessed and the thermodynamic solubility of the stable MO_x_ phase was modeled in order to get insights in material aging, S-absorption capacity and sulfidation mechanisms.

## Materials and methods

### Synthesis of MO_x_/C S-absorbents

**Figure 1:**
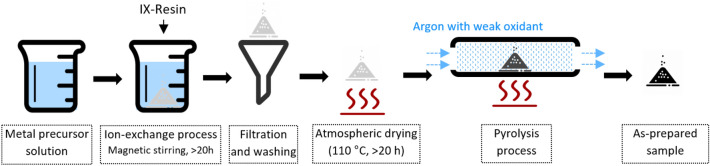
Synthesis flow diagram of MO_x_/C S-absorbent.

The MO_x_/C material preparation method was inspired by Sharma et al.^20^. The method was adjusted to be applied to various metals (M = Zn, Cu, Fe, Mn, Ce) and to obtain higher porosity and stability (pyrolysis under CO_2_ or H_2_O partial pressure). Materials were synthesized generally via two steps: ion exchange followed by pyrolytic carbonization, as illustrated in Fig. 1. The loading of M (or MO_x_) in the carbon matrix relies on the thermodynamics of ion exchange between protons from the carboxylic acid cation exchange resin and M precursors in the first step. Since carboxylic acids are weak acids with small ionization constants, neutral to alkaline media facilitates the cation exchange process. For this purpose, ammonium hydroxide was used to regulate the pH, which specifically made Zn and Cu more soluble in alkaline solution by forming [Zn(NH_3_)_4_]^2+^ and [Cu(NH_3_)_4_]^2+^ complex ions. However, Fe, Mn, and Ce could not form soluble ammonia complexes, their solubility being low in alkaline conditions (hydroxide precipitation). To achieve high M loading, the synthesis methods of different MO_x_/C materials varied according to the solubility of M.

#### ZnO/C and CuO_x_/C

A 0.2 mol L^−1^ Zn^2+^ or Cu^2+^ precursor solution was prepared by dissolving zinc acetate dihydrate (98.0–101%, Alfa Aesar) or copper acetate monohydrate (98%, Alfa Aesar) into de-ionized (DI) water, followed by adding ammonium hydroxide solution (28–30%, Sigma) to reach a pH of 10. The ion exchange resin (DIAION WK 11, Mitsubishi Chemical) used has the carboxylic acid group (-COOH) as the exchange group, with the capacity of 3.0 meq mL^−1^_resin_ and a measured volumetric weight of 0.453 g mL^−1^, which corresponds to 6.623 mmol g^−1^. The resin was then weighed and added to the alkaline [Zn(NH_3_)_4_]^2+^ (or [Cu(NH_3_)_4_]^2+^) solution, with a ratio of M/COOH to be 0.3 (mol mol^−1^). The cation exchange process was performed at room temperature (ca. 20 °C) under gentle agitation (400 rpm) for more than 20 h. The material was then washed with DI water at least three times and dried at 110 °C in air to obtain a dry Zn-resin (or Cu-resin) material. Resin carbonization was then carried out by pyrolysis in a tubular quartz reactor with a diameter of 60 mm under Ar atmosphere (99.99 vol.% purity). The Ar flow rate was controlled at about 80 mL min^−1^ to treat the 3 g M-resin sample. The effect of the presence of a weak oxidant during pyrolysis on the material properties was studied using 1.4 vol.% H_2_O in Ar (saturation at 18 °C and 150 kPa) and 1.0 vol.% CO_2_ in Ar (99.99 vol.% purity of Ar). The quartz tube was heated up to 500 °C at 10 °C min^−1^ and kept for 5 h before cooling down. The as-prepared ZnO/C (or CuO_x_/C) had a spherical and granular shape with a diameter of 0.2–0.4 mm, which volumetrically shrunk about 50% compared to the pre-carbonized Zn-resin (or Cu-resin).

#### FeO_x_/C, MnO_x_/C, and CeO_x_/C

Fe^2+^, Mn^2+^ and Ce^3+^ ions have low solubility in alkaline solution due to their precipitation as hydroxides. Moreover, they could not be converted to soluble complexes by coordination with ammonia. Thus, the pH of their precursor solutions could not be increased to a value above 8, but was limited by the starting point of the precipitation when increasing the pH. Iron(II) acetate (95%, Sigma-Aldrich), Manganese(II) acetate (tetrahydrate, 99%, Sigma-Aldrich), and Cerium nitrate (hexahydrate, > 99%, Fluka) were used as the precursors of Fe, Mn, and Ce respectively. Fe(II) and Mn(II) precursors were chosen for their solubility in higher pH solutions as opposed to their counterparts with higher oxidation states. The initial pH of the 0.2 mol L^−1^ solutions were 6, 7.5, and 4 for Fe^2+^, Mn^2+^, and Ce^3+^, respectively. NH_3_ was added to the three metal solutions dropwise to adjust the pH to 8, 7.5, and 6, respectively, which was the maximum value allowed without precipitation. The following ion exchange and pyrolysis processes were the same for Fe, Mn, and Ce as with Zn. An inert atmosphere (N_2_) was used for Fe and Mn during the ion exchange to avoid the oxidation of their divalent cations.

#### Reference porous C

To investigate the effect of the carbon support, a reference porous C was also tested as a control. The preparation of this C material was carried out by acid washing of the as-prepared ZnO/C illustrated in “ZnO/C and CuO_x_/C” section, allowing full dissolution of the ZnO. In detail, for preparing 0.5 g of carbon material, 1.72 g of ZnO/C (71 wt.% ZnO) was dispersed in 0.5 L 0.1 M HCl solution at room temperature with mild stirring (200 rpm) for 24 h. After separation from the acid by filtration, the spherical particles were washed with plenty of DI water (5 × 1 L) to remove the remaining  Cl^−^ and then dried at 110 °C to obtain the as-prepared porous C.

### Characterization of MO_x_/C S-absorbents

The specific surface area (SSA) and pore structure of fresh and spent MO_x_/C S-absorbents were determined by measuring the N_2_-physisorption (77 K) isotherm using a Quantachrome Autosorb AS1 instrument. The samples were outgassed in a dynamic vacuum for a minimum of 5 h at 250 °C. Brunauer–Emmett–Teller (BET) theory was applied to determine the SSA. The total pore volume was determined at relative pressures p p_0_^–1^ ≥ 0.99. The micropore volume (V_MP_) was determined by the t-method developed by Lippens and de Boer^22^. The non-micropore volume is defined here as the external volume (V_Ext_) and is calculated by subtracting the micropore volume from the total pore volume: V_Ext_ = V_Tot_ − V_MP_. The external surface area (SSA_Ext_) that defines the sample surface area without the micropore contribution was calculated by the same method. The pore diameter distribution and the mean pore diameter were calculated using the adsorption branch based on the Barrett–Joyner–Halenda (BJH) method.

The crystalline phase structure of the solid materials was determined by powder X-ray diffraction (XRD) on a Bruker D8 Advance diffractometer equipped with Ni-filtered Cu Kα-radiation (λ = 1.5418 Å). The diffraction spectrum was collected in the 2θ range from 5° to 90° with the acquisition interval of 0.03° per every 4 s.

To study the material morphology, particle size and shape, transmission electron microscopy (TEM) was performed on two microscopes: a JEOL JEM 2010 microscope operated at 200 keV with a LaB_6_ electron source equipped with a slow-scan CCD camera (4008 × 2672 pixels, Orius Gatan Inc.) and a probe corrected JEOL JEM-ARM200F (NeoARM) microscope equipped with a cold field emission gun operated at 200 keV and a OneView CMOS camera (IS-version; Gatan). The latter microscope was also used for STEM and EDX investigations, for which a JEOL EDX detector “Centurio 100” was used to measure the elemental composition. The particle size of MO_x_/C in TEM images was measured using ImageJ software, 100 particles were randomly selected from representative images.

Quantitative elemental analysis of solid and liquid samples was performed on an ICP-OES device (Agilent 715). The analyzer was calibrated with different dilutions of a multi-element standard solution prepared from certified standards. The standards of the individual elements were purchased either from Ultra Scientific or from Merck. Liquid and solid samples were all pre-treated by digestion in acid containing 5 mL 65% HNO_3_ and 1 mL 37% HCl.

### Evaluation of MO_x_/C S-absorbents under HTG conditions

The performance of MO_x_/C materials was evaluated by using an unstirred 316 stainless-steel batch reactor (BR) with 28 mL volume, assembled using components from HiP. More details of the reactor setup were given in previous work^23^. A preheated fluidized sand bath (IFB51, Techne) was used to quickly heat the BR to the targeted temperature of 450 °C within ca. 15 min. The total volume of the water-based feed was always set at 6.7 mL, in order to reach 30 MPa at 450 °C. The reaction time for all the experiments was kept at 15 min from when the BR temperature reached 450 °C. After each BR test, the reactor was quenched by immersion into a cold-water bath until reaching room temperature. Then, the BR was depressurized and the discharged gas was sampled in a 3 L gas bag (Multi-Layer Foil, RESTEK) for immediate offline gas analysis using a calibrated µGC (Micro GC Fusion, INFICON). Then, 4 mL of isopropanol was added to the opened BR to dissolve the water-insoluble organics (solids, liquid organic phase), allowing the sampling of a homogeneous liquid. The sampling of spent solids was carried out by direct separation from the liquid, followed by washing the particles with ethanol and water (three times each) before drying at 110 °C.

To study the individual effect of pure SCW on MO_x_/C (M = Zn and Cu) materials, an “SCW-stability” test was carried out by exposure of 100 mg MO_x_/C (M = Zn and Cu) to 6.7 mL DI water in the BR and maintaining the HTG conditions of 450 °C and 30 MPa for 24 h.

The sulfur absorption performance of the MO_x_/C materials under HTG conditions was evaluated by using aqueous hydrocarbon as the feed to mimic the wet biomass and using model organoS (organic sulfur compound) as the S source. To evaluate the capacity of MO_x_/C materials to absorb sulfur species, an excess of S with regard to M was used. M/S ratio was set to 2 (mol_M_ mol_S_^−1^) considering some metals (e.g. Cu) can form an M_2_S composite with S. S should be in the form of H_2_S or a form leading to the formation of H_2_S in SCW. To that end, diethyl disulfide (DEDS) was used to generate H_2_S in SCW as it is known to have low stability under these conditions and to lead to H_2_S formation in situ^24^. With the BR setup and reaction conditions of 450 °C, 30 MPa, and 15 min, 6.7 mL of model S feed solution (DEDS in a water/isopropanol mix) was used corresponding to an absolute sulfur amount of 0.397 mmol_S_. The MO_x_/C was added to the DEDS solution in a quantity such that the M amount was 0.794 mmol_M_. The amount of C tested was set at 100 mg to keep a similar total weight as with the porous MO_x_/C (M = Zn, Fe and Cu). To validate the reproducibility of such S-absorption tests, the tests without solid material (“solution alone”), with ZnO/C, and with CuO_x_/C were repeated with a coefficient of variation of less than 6.5%. The corresponding standard deviation are indicated in Fig. 3. Due to the optimum performance of CuO_x_/C, a “maximum” S-absorption capacity test was individually carried out for it using the same DEDS feed but with different conditions of M/S = 1 mol_M_ mol_S_^−1^ with an HTG reaction time of 15 h.

### The analysis of sulfur compounds

The total amount of S in either liquid phase or solid phase was determined by ICP-OES with the instrument and sample pretreatment methods described above in “Characterization of MO_x_/C S-absorbents” section. An ion chromatography device (IC, Metrohm) using a Metrosep A Supp 10-100/4.0 column and Metrohm 732 IC conductivity detector was used to quantify the sulfate ions in liquid samples.

Light organosulfur compounds in the liquid residue were analyzed by gas chromatography (GC, Agilent 7890A) using an Agilent J&W DB-Sulfur SCD column (40 m × 0.32 mm ID) with helium (quality 6.0) as the carrier gas. A sulfur chemiluminescence detector (SCD, Agilent 355) with a dual plasma burner was coupled for the selective analysis of S-containing organic compounds^25^. An identical column equipped in parallel and connected to a flame ionization detector (FID) was used to give general information on the presence of other (S-free) organic compounds. The oven temperature was programmed as 40 °C for 7 min before rising at 7 °C min^−1^ up to 220 °C for 8 min. Theoretically, the quantitative calibration of sulfur compounds can be based on the “equimolar response” of SCD^26^. Dibenzothiophene was used as an internal standard to perform a quantitative analysis of volatile sulfur compounds. The sum of all the organosulfur calculated by this semi-quantitative method is always higher than the total amount of S in liquid phase measured by ICP-OES. Considering a higher accuracy of the latter, the amount of organosulfur compounds was proportionally corrected based on the total S measured by ICP-OES. The retention time (RT) of S compounds was determined by testing various pure organosulfur standards, including methanethiol, dimethyl sulfide, dimethyl disulfide (DMDS), dimethyl trisulfide (DMTS). Thanks to the column and method used, a linear relationship existed between the boiling point (BP) and RT and is given in Fig. S1, so that the BP of an unknown sulfur-containing compound could be extracted from the RT.

### Thermodynamic modeling of MO_x_ dissolution in water

The reaction equations between MO_x_ (ZnO, Cu_2_O, Cu, Fe_3_O_4_, MnO, and CeO_2_) and H_2_O, listed in Table S2, to form the corresponding metal ions or hydrated metal ions, were used to model the dissolution of MO_x_ in pure neutral water. Redox reactions of ZnO, Cu_2_O, Fe_3_O_4_, MnO, and CeO_2_ were not considered because of their stable oxidation states under HTG conditions, proven by the material characterizations in the experimental work of this study. Only the dissolution of Cu(0) was considered to be a redox reaction with the formation of Cu^+^ and aqueous H_2_. To simplify the model, Cu^+^ was regarded as the preferential dissolution product of Cu(0), Cu^2+^ being excluded due to the lower thermodynamic barrier for its formation. Eight different conditions (*T*, *P*, *ρ*_H__2__O_) were modeled, as listed in Table S4, corresponding to different states of water from liquid to supercritical, which were extracted from a representative *T*&*P* profile (see Fig. S12) in the experimental work of this paper. Following the revised Helgeson–Kirkham–Flowers (R-HKF) thermodynamic model applied by Shock et al.^27^ and Jocz et al.^16^, equilibrium constants (*K*_*eq*_) of the MO_x_ dissolution reactions listed in Table S2 were firstly calculated. Secondly, concentrations of dissolved M ions or hydrated M ions in water as multiple unknowns were solved using Matlab. All the calculation details are given in the Appendix of the Supplementary Information.

Briefly, *K*_*eq*_ was calculated from the change in Gibbs free energy of the dissolution reactions (*ΔG*_*rxn*_) as shown in Equation S1, where the calculation of *ΔG*_*rxn*_ was based on the apparent standard partial molar Gibbs free energy of the formation ${\Delta G}_{f}{\left(T,{\rho }_{{H}_{2}O}\right)}_{j}$ of each species (*j*) involved in the reaction and their stoichiometric coefficient (${v}_{j}$), given in Equation S2. Depending on the phase of *j*, ${\Delta G}_{f}{\left(T,{\rho }_{{H}_{2}O}\right)}_{j}$ was calculated using the specific expression.

${\Delta G}_{f}{\left(T,{\rho }_{{H}_{2}O}\right)}_{j}$ of solid *j* and H_2_O under a targeted condition (*T, P, ρ*_*H2O*_) was calculated using the differential expression for apparent standard partial molar Gibbs free energy (Equation S3). The thermodynamic parameters at the standard reference temperature (*T*_*0*_ = 298.15 K) and pressure (*P*_*0*_ = 1 bar) for such calculation, such as the standard partial molar Gibbs free energy of formation of *j* (*ΔG*_*j*_^*0*^) from the elements in their stable form, and the standard molar entropy (*S*_*j*_^*0*^) were obtained from the thermochemical database^28,29^. Table S3 summarizes those standard thermodynamic parameters of MO_x_ solid species. Table S4 lists the calculated thermodynamic and solvent-related parameters of water at the selected eight different conditions.

${\Delta G}_{f}{\left(T,{\rho }_{{H}_{2}O}\right)}_{j}$ of ions and dissolved H_2_ and O_2_ was calculated using the R-HKF equation^27^ (Equation S5). The standard thermodynamic and HKF parameters (species-dependent non-solvation parameters) of specific aqueous ions were obtained from Shock et al.^30^ and are reprinted in Table S5. ${\Delta G}_{f}{\left(T,{\rho }_{{H}_{2}O}\right)}_{j}$ of H^+^ was set as the reference for all aqueous species and equaled to 0 at all conditions, while ${\Delta G}_{f}{\left(T,{\rho }_{{H}_{2}O}\right)}_{j}$ of OH^−^ was calculated using Equation S14.

Therefore, based on the Equations S2–13, *ΔG*_*rxn*_ and *K*_*eq*_ of the targeted dissolution reactions of MO_x_ under the eight different states of water were calculated as listed in Table S6. To further calculate the molar concentrations *m*_*j*_ (mol kg^−1^) of all the dissolved species in water at a specific condition, Equation S15 relating the equilibrium constants (*K*_*eq*_) to the thermodynamic activity coefficient (*γ*_*j*_) was used, where *γ*_*j*_ could be correlated to the ionic strength (*I*) and the Debye-Hückel parameter (*A*_*Φ*_) using Davies extension of the Debye-Hückel equation^31^ in Equation S16. Assuming N total aqueous species (H^+^, OH^−^, and N-2 dissolved ions from the MO_x_) are existing in water, there are N + 1 unknown variables (N concentrations *m*_*j*_ plus ionic strength* I*). Combining the N-2 equilibrium expressions (Equation S15) for all dissolution reactions, the definition of the ionic strength (*I*) of the solution (Equation S18), the equilibrium equation for the ion product of water, and the expression of charge neutrality (Equation S19), N + 1 equations were set up to solve the N + 1 unknowns. Matlab’s nonlinear least-squares solver function (lsqnonlin) with the “Levenberg–Marquardt” algorithm was used to solve the equations and ultimately calculate a total concentration of dissolved compounds.

## Results and discussions

### Synthesis development and characterization of MO_x_/C materials

Aiming at high MO_x_ loadings in the MO_x_/C composites as well as a uniform distribution of MO_x_ in the C matrix, the synthesis process was optimized in terms of ion exchange and pyrolytic carbonization as illustrated in Fig. 1.

#### The optimization of pyrolysis conditions in ZnO/C synthesis

In a previous study, pure Ar was used for the pyrolytic carbonization of the methacrylic acid resin^32^. For optimum sulfur absorption performance, one should aim at increasing the porosity of the MO_x_/C material and increasing MO_x_ loading (more C oxidized). For that, weak oxidants, i.e. H_2_O and CO_2_, were added to Ar in the pyrolysis process. Steam or CO_2_ co-feeding with the inert gas stream was proved in biomass pyrolysis to enhance the porosity of the biochar^33^. This treatment with steam or CO_2_ supposedly also favors the formation of phases with higher stability in supercritical water.

Figure S2 presents the XRD patterns of two ZnO/C materials synthesized with 1.4 vol.% H_2_O in Ar and 1 vol.% CO_2_ in Ar. They present very similar crystalline composition, all diffraction peaks could be assigned to hexagonal ZnO. Similar peak intensity was also observed, and the average ZnO crystallite size according to the Scherrer Equation was found to be 22 nm and 21 nm for “CO_2_ in Ar” and “H_2_O in Ar”, respectively.

Different pyrolysis temperatures (400, 500, and 600 °C) were also studied with the H_2_O- and CO_2_-containing Ar. A control test with pure argon at 500 °C was also performed to investigate the effect of water or carbon dioxide. The ZnO loading and the pore properties of the ZnO/C materials obtained under different pyrolysis conditions are summarized in Table 1.

**Table 1:** The effect of pyrolysis conditions on the properties of ZnO/C materials.

Pyrolysis gas	Temperature (°C)	Sample appearance	ZnO content (wt%)	BET surface area (m^2^ g^−1^)	Total pore volume (cm^3^ g^−1^)
Dry Ar	500	Homogeneous/black	48	272	0.19
1.4 vol.% H_2_O in Ar	400	Homogeneous/brownish	52	27	0.10
	500	Homogeneous/black	67	255	0.16
	600	Heterogeneous/black and white	77	301	0.21
1 vol.% CO_2_ in Ar	400	Homogeneous/brownish	47	7	0.07
	500	Homogeneous/black	58	277	0.20
	600	Heterogeneous/black and white	63	293	0.18

As shown in Table 1, under the same pyrolysis temperature of 500 °C, the presence of H_2_O or CO_2_ in the Ar increases the ZnO content in the as-prepared ZnO/C. In parallel, the specific surface area and pore volume seem to be lower. With H_2_O/Ar or with CO_2_/Ar, the ZnO content increased with the pyrolysis temperature, indicating the gasification of carbon. Eventually, at the highest temperature tested, i.e. 600 °C, the material shows a heterogeneous appearance with the presence of white particles among the black/grey particles, indicating a nearly complete gasification of the carbon in some area of the material bed. Even though 600 °C produced the highest surface area, pore volume and loading both with H_2_O/Ar and CO_2_/Ar, the heterogeneity of the MO_x_ distribution in the material led to choose 500 °C under H_2_O/Ar atmosphere as optimum pyrolysis conditions. The use of H_2_O leads to a loading 9 wt% higher than when CO_2_ was used, while the pore volume are > 0.15 cm g^−1^ in both cases. The optimal pyrolysis condition was therefore defined as 500 °C and 1.4 vol.% H_2_O in Ar. These conditions were used for the synthesis process of all other MO_x_/C materials.

#### Characterization of the as-prepared MO_x_/C materials

As shown in Table 2, both ZnO/C and CuO_x_/C reached a high metal loading with > 0.3 mol_M_ mol_C_^−1^ (71 and 62 wt%, respectively), due to the high solubility of metal cations in alkaline solution. The maximum pH allowed before precipitation for Fe^2+^, Mn^2+^, and Ce^3+^ solutions was 8, 7.5, and 6, respectively. Higher pH is favored during the ion exchange step in the MO_x_/C synthesis. Thus their MO_x_ loadings follow the order of FeO_x_/C (56.4 wt%) > MnO_x_/C (50.0 wt%) > CeO_x_/C (27.3 wt%). Their crystalline phases were analyzed by XRD (Fig. S4) and are summarized in Table 2. ZnO/C, FeO_x_/C and CeO_x_/C present single-phase MO_x_ crystalline grain, which is ZnO (hexagonal), Fe_3_O_4_ (cubic), and CeO_2_ (cubic), respectively. Differently, CuO_x_/C and MnO_x_/C both show more than two crystalline phases of metal oxides. For CuO_x_, a mixture of Cu_2_O (cubic) and Cu (cubic) is observed according to the XRD pattern (Fig. S4b), which is different to the pure Cu(0) phase obtained by using dry Ar to pyrolyze [Cu(NH_3_)_4_]^2+^ exchanged methacrylic acid-based resin^32^. MnO_x_ (Fig. S4d) shows the main crystalline phase as MnO (cubic) with small amounts of Mn_2_O_3_ (orthorhombic).

**Table 2:** Properties of the various as-prepared MO_x_/C and C.

		ZnO/C	CuO_x_/C	FeO_x_/C	MnO_x_/C	CeO_x_/C	C^a^
Main crystalline phases (from XRD)		ZnO	**Cu**_**2**_**O**^b^/Cu	Fe_3_O_4_	**MnO**^b^/Mn_2_O_3_/	CeO_2_	n.d.^c^
Corresponding Tammann temperature^d^	°C	851	481 (Cu_2_O) 406 (Cu)	646	836 (MnO) 333 (Mn_2_O_3_)	1063	N/A^e^
MO_x_ content^f^	wt%	71.0	61.8	56.4	50.0	27.3	< 0.1
M/C in material	mol mol^−1^	0.36	0.30	0.20	0.17	0.03	< 0.01
Total SSA	m^2^ g^−1^	235	244	200	3	1	1077
Micropore SSA	m^2^ g^−1^	192	181	48	< 1	< 1	595
Total pore volume	cm^3^ g^−1^	0.17	0.14	0.18	0.01	0.02	1.00
Micropore volume	cm^3^ g^−1^	0.08	0.07	0.02	< 0.01	< 0.01	0.25
Mean pore diameter^g^	nm	2.9	2.3	3.6	10.4	128	3.7
Average crystallite size (by XRD)	nm	19	47 (Cu_2_O) 53 (Cu)	20	45 (MnO) 46 (Mn_2_O_3_)	4	N/A
Particle size (from TEM)^h^	nm	14 ± 5	26 ± 7	15 ± 4	20 ± 17	< 5	N/A

^a^Prepared by acid washing of ZnO/C.

^b^The bold text represents the main MO_x_ crystalline phase present.

^c^Not detected.

^d^Tammann temperature corresponds to half of the melting point in degrees Kelvin^35^.

^e^Not applicable.

^f^Determined by ICP analysis.

^g^Determined by Barrett-Joyner-Halenda (BJH) method on the adsorption branch.

^h^The average size with standard deviation of 100 random particles from representative TEM images (Fig. 2) measured using ImageJ software.

**Figure 2:**
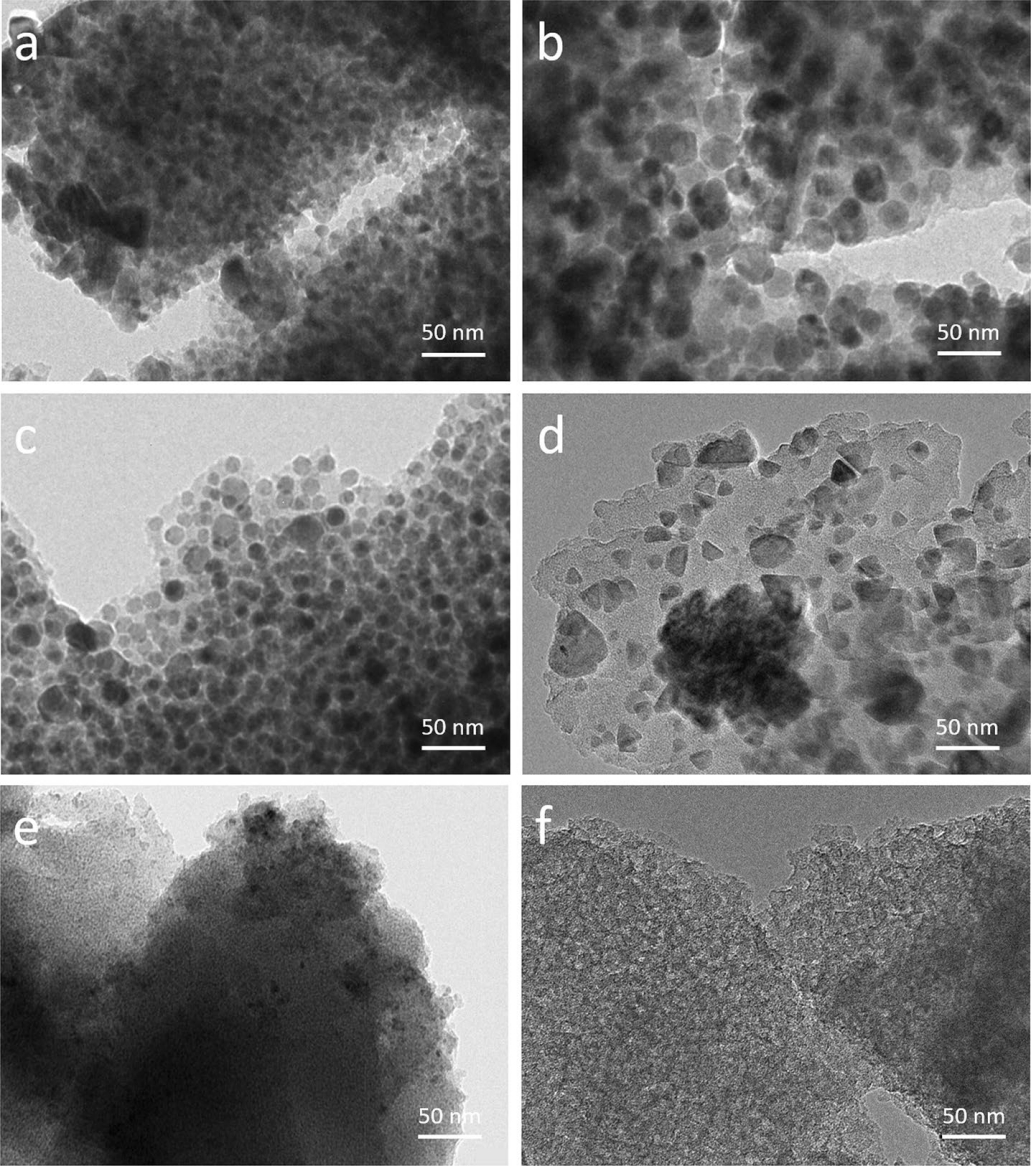
TEM images of as-prepared (**a**) ZnO/C, (**b**) CuO_x_/C, (**c**) FeO_x_/C, (**d**) MnO_x_/C, and (**e**) CeO_x_/C using the pyrolysis conditions of 500 °C and 1.4 vol.% H_2_O in Ar. (**f**) TEM image of as-prepared C by acid treatment of ZnO/C.

The as-prepared materials all showed nano-scale particles uniformly dispersed in the carbon matrix as evidenced by TEM in Fig. 2. The particle size distribution (PSD) varied by the type of M, which were evaluated from TEM images and are plotted in Fig. S5. From the representative images given in Fig. 2a–e, the particle size of ZnO, CuO_x_, FeO_x_, MnO_x_, and CeO_x_ embedded in C is in the range of 14 ± 5, 26 ± 7, 15 ± 4, 20 ± 17, and < 5 nm, respectively, following the order CuO_x_ > MnO_x_ > FeO_x_ ≈ ZnO >  > CeO_x_. Their crystalline phases and corresponding crystallite size determined by XRD (in Fig. S4) are ZnO (19 nm), Cu_2_O (47 nm)/Cu (53 nm), Fe_3_O_4_ (20 nm), MnO (45 nm)/Mn_2_O_3_ (46 nm), and CeO_2_ (4 nm) indicating overall that crystalline particles are larger than the average. The different particle and crystallite size of specific MO_x_ formed might be explained by Tammann temperatures (summarized in Table 2) that correlate to a material resistance towards sintering^34^. ZnO, FeO_x_, and CeO_x_ present finer and more uniform particle sizes than CuO_x_ and MnO_x_ thanks to the higher Tammann temperatures and purer oxidation states. TEM micrograph of the control C material prepared by acid washing of ZnO/C is shown in Fig. 2f. The absence of particles supports the ICP analysis in that no Zn remained after the acid washing and indicates a high purity of C.

N_2_ adsorption–desorption isotherms (Fig. S3) were used to characterize the pore structure of MO_x_/C and C with the results summarized in Table 2. ZnO/C, CuO_x_/C, and FeO_x_/C all show porous properties with SSA higher than 200 m^2^ g^−1^ and total pore volume over 0.14 cm^3^ g^−1^. Their isotherms indicate mainly type I/II with little to no hysteresis indicating an open pore structure with some microporosity^36^. They have a broad pore size distribution, as presented in the inserts of Fig. S3a–c. ZnO/C, CuO_x_/C, and FeO_x_/C have a microporosity of 47%, 50%, and 11% of the total pore volume, respectively. The mean pore size of the three materials is in the range of 2–4 nm, which is much smaller than the particle size of MO_x_ embedded into the carbon matrix.

The C material prepared by acid washing of ZnO/C shows a specific surface area of 1077 m^2^ g^−1^ and a total pore volume of 1.0 cm^3^ g^−1^. Its isotherms (see Fig. S3f) present a continuously increasing adsorption branch in line with a broad pore size distribution, but a very sharp drop of adsorbed gas at P/P_0_ = ca. 0.47 on the desorption branch, which is typical of mesopores connected to the outer surface through micropores (ink-bottle-shaped pores). The pore size distribution obviously indicates two pore families: micropores (volume 0.25 cm^3^ g^−1^) and mesopores mainly distributed around 8 nm. The TEM image (see Fig. 2f) points to a very homogeneous porous material with a high porosity in line with a dissolution of ZnO particles yielding mesoporous cavities. The ink-bottle-shaped pores confirm that the ZnO/C material consists mainly of ZnO particles embedded by microporous carbon, which after dissolution in acid leaves cavities connected to the outer surface through micropores.

When metal-free resin was pyrolyzed, the product obtain consisted of non-porous carbon block/sheets, indicating that the polymer melted before pyrolysis. In all cases, the metal content impacted the resin melting/pyrolysis as all MO_x_/C consisted of spheres of the same diameter as the starting resin. However, MnO_x_/C and CeO_x_/C show poor porosity with a specific surface area below 3 m^2^ g^−1^. This indicates that the two elements do not impact the decomposition of the polymer the way Zn, Cu, Fe or Ni do, maybe because the salts decompose and the oxides form after the polymer melted and started to pyrolyze^20^. In the case of Ce, the low pore volume and surface area might also be explained by the lower loading of Ce in the material.

### The S-absorption capacity and efficiency of MO_x_/C materials

**Figure 3:**
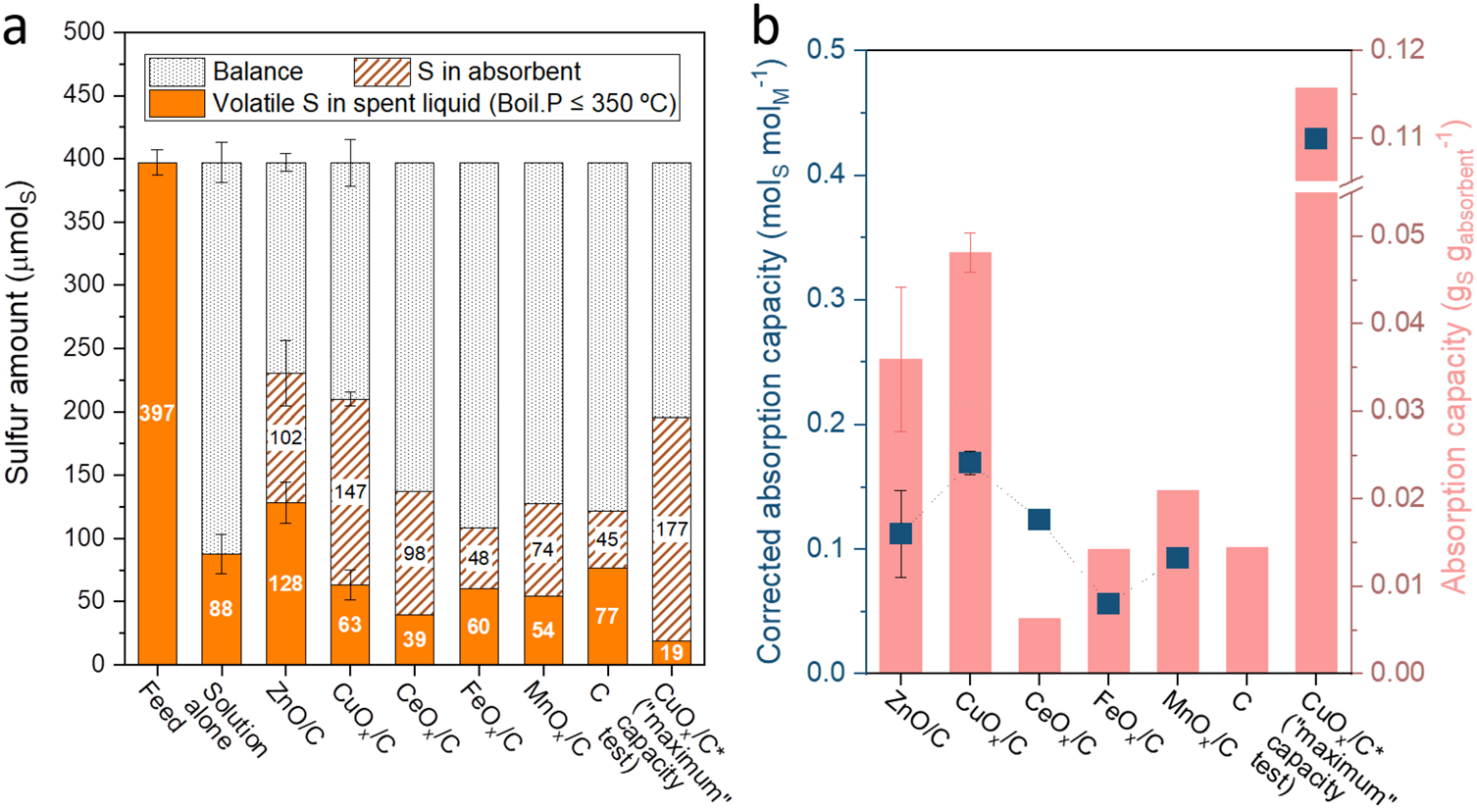
(**a**) Distribution of S by class of compounds in the DEDS feed and after HTG sulfur absorption capacity tests without (solution alone) and with MO_x_/C and C materials. The sulfur balance might end up in the gas phase or as organic solids insoluble in the isopropanol-water mixture. *- “maximum” S-absorption capacity test using the same DEDS feed but with different conditions of M/S = 1 mol_M_ mol_S_^−1^ and HTG reaction time 15 h instead of 15 min. (**b**) The S-absorption capacities of MO_x_/C and C materials in different units (mol_S_ mol_M_^−1^ and g_S_ g_absorbent_^−1^).

The sulfur absorption capacity of MO_x_/C materials was tested using an M/S ratio of 2 mol_M_ mol_S_^−1^ for 15 min, but also with 1 mol_M_ mol_S_^−1^ for 15 h (CuO_x_/C, marked with an asterisk). Diethyl disulfide (DEDS) in a water/isopropanol mix was used as the source of sulfur, with an absolute S amount of 397 µmol_S_. This model organosulfur compound readily decomposes into H_2_S in supercritical water along with a small amount of ethanethiol^24^. DEDS and MO_x_/C were loaded into the BR for the HTG reaction (450 °C, 30 MPa, and 15 min). After the reaction, the gas product was analyzed by a microGC with the composition shown in Fig. S6a. Gaseous sulfur species (mostly H_2_S and partially ethanethiol^24^) were not determined due to the presence of overlapping water and the low sensitivity of the microGC, respectively. The amount of sulfur in the DEDS feed and in the spent solid and liquid was determined and the balance results are shown in Fig. 3a.

As illustrated in Fig. 3a, out of the 397 µmol_S_ of DEDS in the feed, after cHTG reaction without S-absorbents (solution alone), about 22% of the sulfur remained in solution as volatile organoS. A small amount (ca. 15 µmol_S_) of SO_4_^2−^ was also detected in all the spent liquids but most likely originated from the oxidation of dissolved H_2_S by air in the aqueous solution upon sample preparation^37^. The feed only contains two organic compounds, i.e. isopropanol ((CH_3_)_2_CHOH) and DEDS (C_2_H_5_SSC_2_H_5_), SCW should break down most C–C and C-S bonds such that ethanethiol (C_2_H_5_SH) should be the main remaining organoS in all spent liquids^38^.

Identified and semi-quantified by GC-SCD (see Fig. S6b), the liquid phase after testing was mainly made up of volatile sulfur compounds (BP ≤ 350 °C). Without using S-absorbent, the spent liquid was mainly composed of 60 µmol_S_ ethanethiol and 15 µmol_S_ DEDS. Note that the DEDS observed might be formed back from ethyl thiyl radicals during the quenching of the reactor, or from ethanethiol after sampling^19^. Other compounds such as isopropyl disulfide ((CH_3_)_2_CHSSCH(CH_3_)_2_), methyl ethyl trisulfide (CH_3_SSSC_2_H_5_), and diethyl trisulfide (C_2_H_5_SSSC_2_H_5_) might also have been formed from corresponding radicals present in the supercritical fluid or from the corresponding thiols and H_2_S after sampling. The presence of different materials in the solution only led to a significant decrease of ethanethiol, their impact on the concentration of other identified volatile organosulfur compounds was marginal except for ZnO/C, which showed a higher concentration of trisulfides.

Comparing the amount of S absorbed in the materials (striped pattern in Fig. 3a), it is interesting to note firstly that the C alone, when exposed to HTG conditions, leads to an accumulation of S (see Fig. 3a,b, “C”). Micropores are known to favor coking and quickly foul during cHTG reaction which might explain the deposition of sulfur on the material in the form of coke, or simply adsorbed inside^39^. Although the porous framework was maintained in the spent C with a total SSA of 599 m^2^ g^−1^ (Fig. S7a), the micropore volume decreased by 64% compared to the fresh C. A comparison of the pore size distribution between the fresh C and spent C directly shows the decreased micropore proportion after the reaction (Fig. S7b) supporting this hypothesis. Accordingly, the S-absorption capacity in mol_S_ mol_M_^−1^ (Table 3 and Fig. 3b, left Y-axis) is a corrected value after subtracting the micropore-absorbed S using the micropore volume of MO_x_/C (Table 2) and the microporous capacity of C (1.81 mmol_S_ cm_micropore_^−3^ calculated from the test of C). The order of S-absorption capacity (mol_S_ mol_M_^−1^) can be summarized as CuO_x_/C > CeO_x_/C ≈ ZnO/C > MnO_x_/C > FeO_x_/C. CuO_x_/C presented the highest S-absorption capacity with 0.17 mol_S_ mol_M_^−1^ and 0.048 g_S_ g_absorbent_^−1^. CeO_x_/C shows a slightly higher absorption capacity than ZnO/C, 0.12 vs. 0.11 mol_S_ mol_M_^−1^ (cf. Table 3), even though the absolute amount of S captured by ZnO/C is higher than CeO_x_/C (Fig. 3a). In the case of ZnO/C, up to 12% of the deposited S might be due to the carbon microporosity alone. The very low surface area and microporosity of CeO_x_/C should have resulted in a negligible amount of S deposition on the carbon itself, which explains, together with the low loading of ceria, the low mass-based S-absorption capacity (only 0.006 g_S_ g_absorbent_^−1^) in Fig. 3b. FeO_x_/C shows 48 µmol sulfur captured, very similar to the C alone, but because FeO_x_/C had five and ten times lower total surface area and micropore volume, respectively, it can be estimated that the material absorbed 0.06 mol_S_ mol_M_^−1^ (see Table 3) after correction for the estimated C absorption. The capacity of MnO_x_/C to absorb S seems also high, taking into consideration its low surface area and the probably negligible impact of its carbon binder on the material performance. A “maximum” S-absorption capacity test was carried out solely on CuO_x_/C under a much longer reaction time (15 h) but with a lower M/S ratio (1 mol_M_ mol_S_^−1^). After this long test, a higher S-absorption capacity was reached, that is 0.43 mol_S_ mol_Cu_^−1^, compared to 0.17 mol_S_ mol_Cu_^−1^ after 15 min with M/S ratio = 2 mol_M_ mol_S_^−1^. The inability of copper to reach full sulfidation might be due to the formation of H_2_S in the gas phase before it can react with the S-absorbent, which, due to the absence of stirring in the reactor, will only slowly diffuse to the supercritical fluid and to the solid material.

**Table 3:** The sulfided form and sulfidation rate of MO_x_/C after HTG sulfur absorption capacity tests. The theoretical S-absorption capacity is based on the metal-sulfur composition of the MS_x_ phase observed by XRD.

	ZnO/C	CuO_x_/C	FeO_x_/C	MnO_x_/C	CeO_x_/C	CuO_x_/C*
Sulfided form of M	ZnS	Cu_1.8_S	n.d.^c^	MnS	Ce_2_S_3_	Cu_1.8_S
Theoretical S-absorption capacity (mol_S_ mol_M_^−1^)	1.00	0.56	N/A^d^	1.00	1.5	0.56
Experimental S-absorption capacity (mol_S_ mol_M_^−1^)^a^	0.13	0.18	0.06	0.09	0.12	0.44
Corrected experimental S-absorption capacity (mol_S_ mol_M_^−1^)^b^	0.11	0.17	0.04	0.09	0.12	0.43
Sulfidation rate (*%*)	11	31	N/A^d^	9.3	8.2	77

^*^“Maximum” S-absorption capacity test with M/S = 1 mol_M_ mol_S_^−1^ and HTG reaction time 15 h.

^a^Dividing the absolute amount of S absorbed in MO_x_/C by the amount of M.

^b^Corrected value by subtracting the micropore-absorbed S, calculated using the equation $\frac{n(S \; absorbed \; in \; M{O}_{x}/C) - n(S \; absorbed \; in \; micropore)}{n(M)}$, where $n(S \; absorbed \; in \; M{O}_{x}/C)$ are the moles of S absorbed in MO_x_/C obtained experimentally, $n\left(S \; absorbed \; in \; micropore\right)$ is the product of MO_x_/C micropore volume (given in Table 2) and the micropore S-absorption capacity of C (1.81 mmol_S_ cm_micropore_^−3^ calculated by the S-absorption capacity test of C). n(M) are the moles of metal used for the test.

^c^Not detected.

^d^Not applicable.

**Figure 4:**
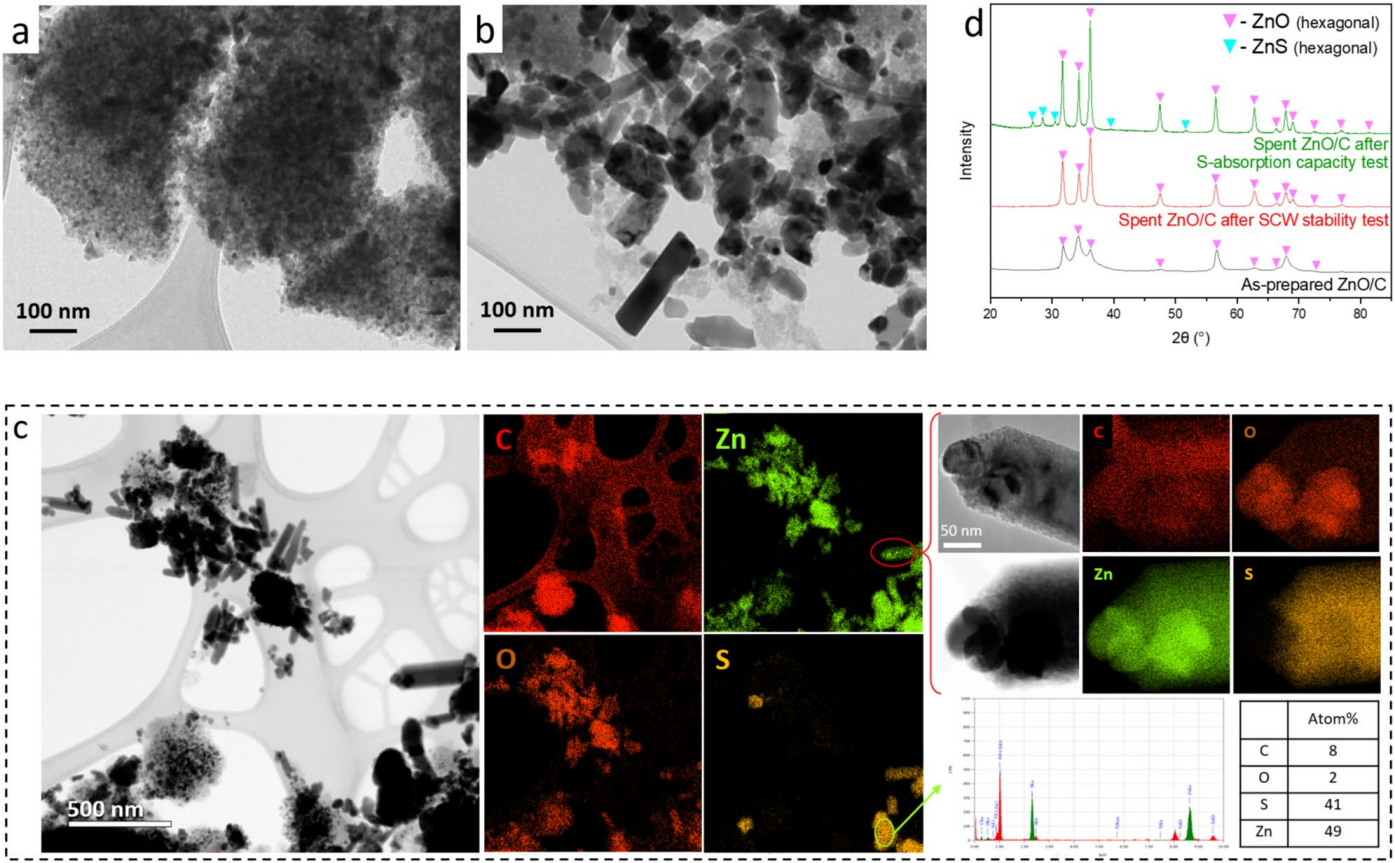
TEM images of (**a**) as-prepared ZnO/C, (**b**) spent ZnO/C after SCW treatment for 24 h under 450 °C and 30 MPa. (**c**) TEM images and STEM-EDX elemental mapping of spent ZnO/C after S-absorption capacity test with DEDS model feed (15 min). (**d**) XRD patterns of these three materials.

**Figure 5:**
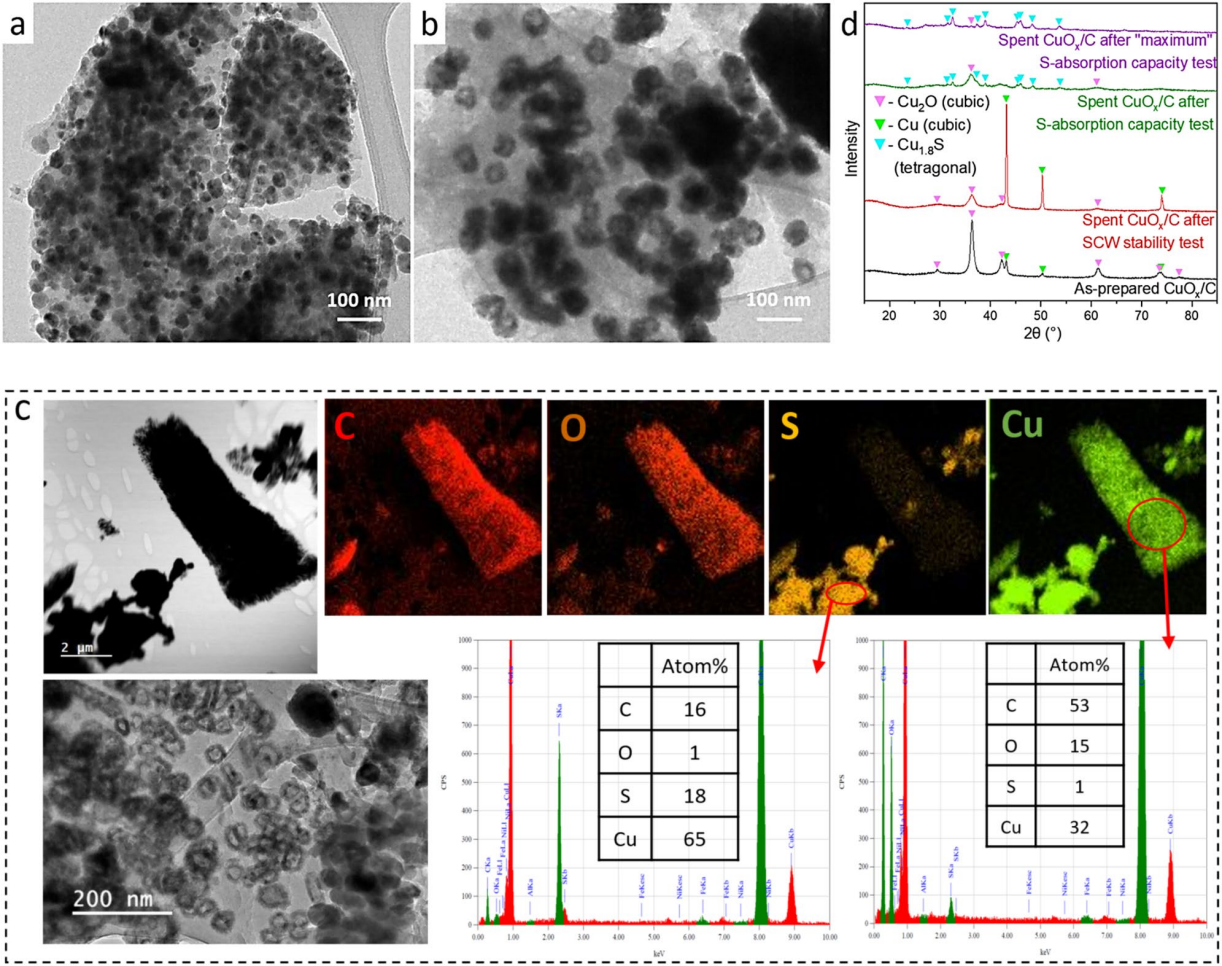
TEM images of (**a**) as-prepared CuO_x_/C, (**b**) spent CuO_x_/C after SCW treatment for 24 h under 450 °C and 30 MPa. (**c**) TEM images and STEM-EDX elemental mapping of spent CuO_x_/C after S-absorption capacity test with DEDS model feed. (**d**) XRD patterns of these 3 materials and the spent CuO_x_/C after “maximum” S-absorption capacity test.

**Figure 7:**
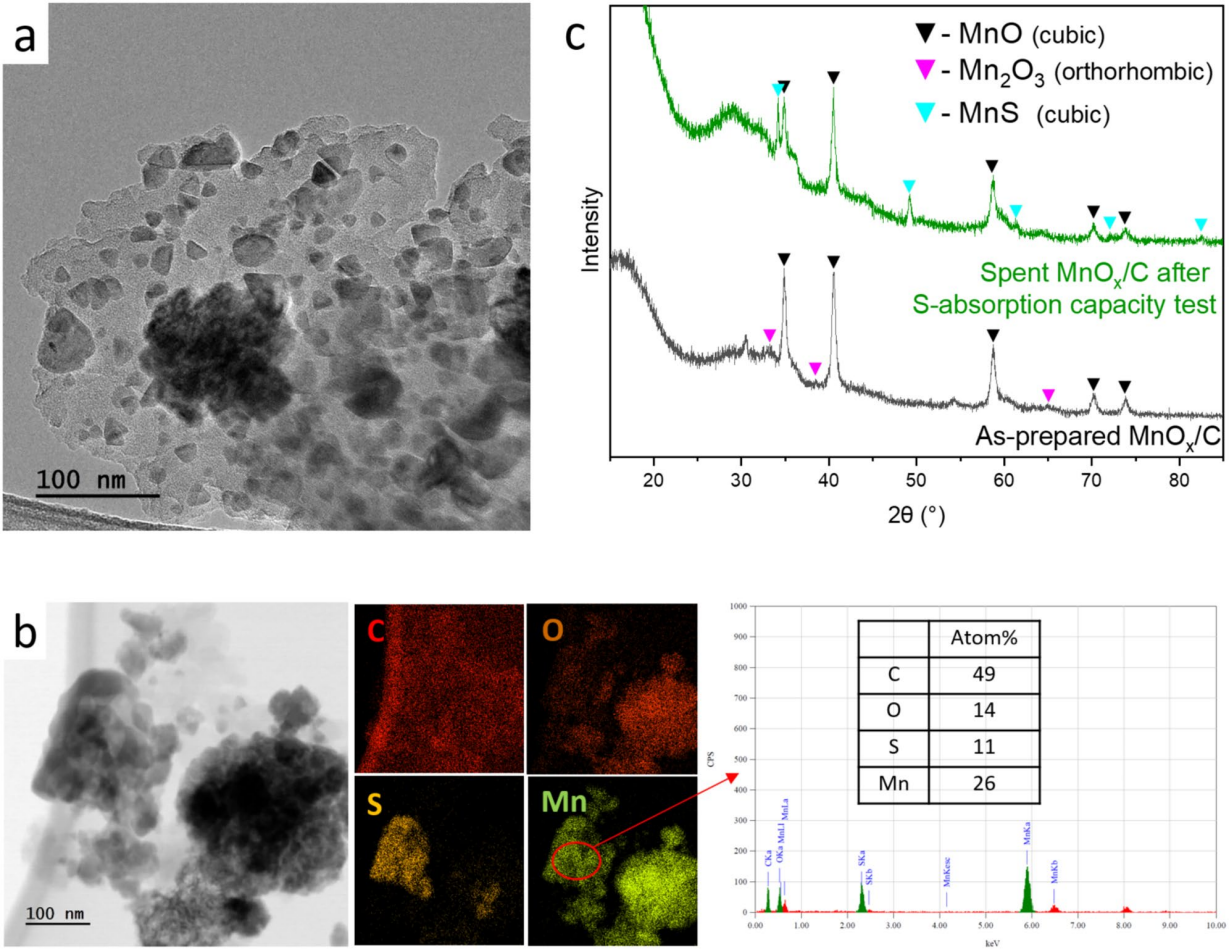
(**a**) TEM image of as-prepared MnO_x_/C. (**b**) TEM image and STEM-EDX elemental mapping of spent MnO_x_/C after S-absorption capacity test with DEDS model feed. (**c**) XRD patterns of these two materials.

**Figure 8:**
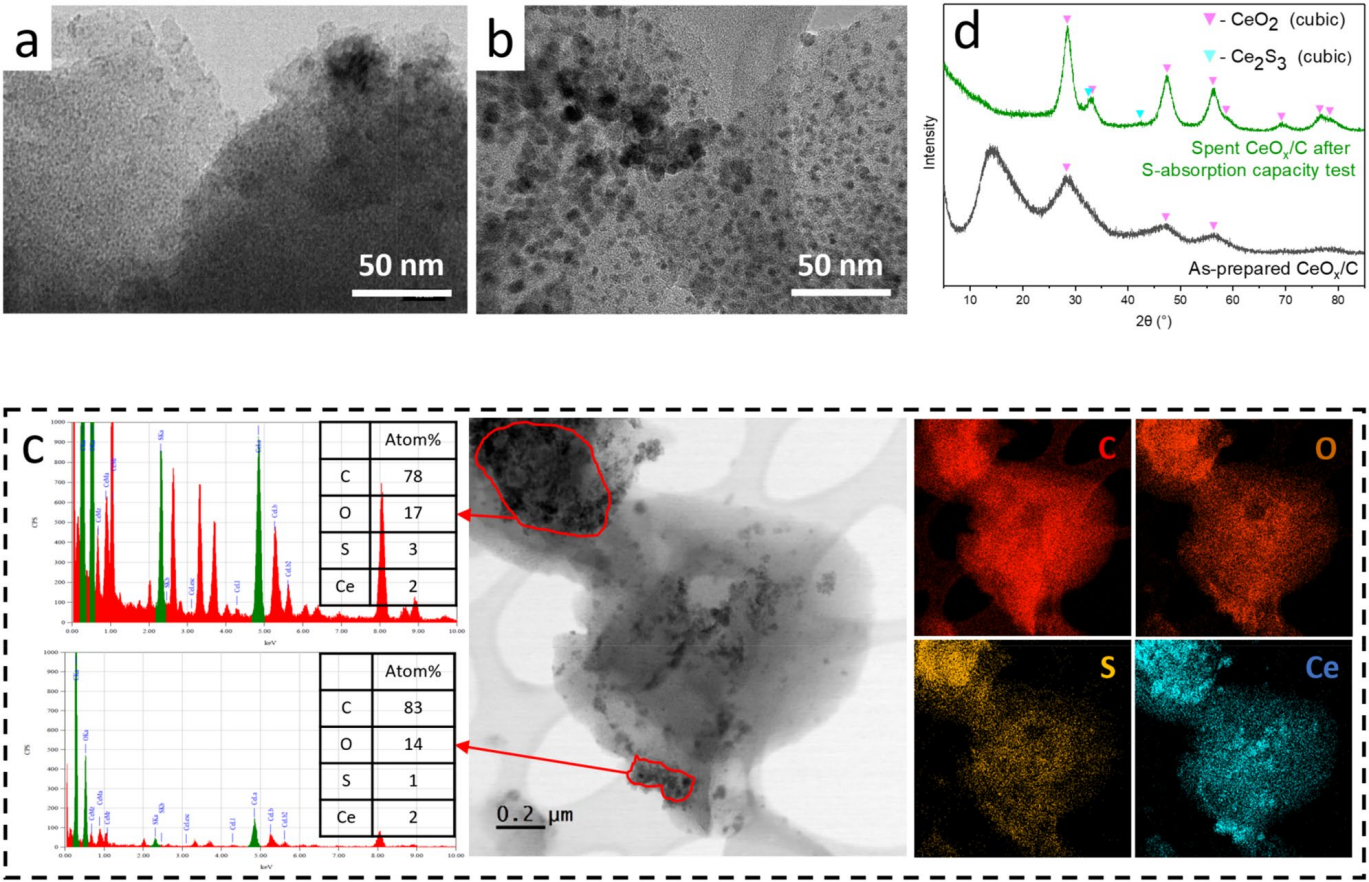
TEM image of (**a**) as-prepared CeO_x_/C. (**b**) TEM image and (**c**) STEM-EDX elemental mapping and HR-TEM image of spent CeO_x_/C after S-absorption capacity test with DEDS model feed. (**d**) XRD patterns of these two materials.

The XRD patterns of MO_x_/C after these sulfur absorption capacity tests are shown in Figs. 4, 5, 6, 7, 8, and are summarized in Fig. S4. The original oxidation state of metals in those MO_x_/C materials was mostly maintained. More specifically, ZnO, Fe_3_O_4_, Cu_2_O, MnO and CeO_2_ were the dominating crystalline phases in the fresh ZnO/C, FeO_x_/C, CuO_x_/C, MnO_x_/C, and CeO_x_/C, respectively, and did not suffer from reduction/oxidation during reductive HTG reaction. The spent MO_x_/C all show crystalline metal-sulfide phases with the exception of FeO_x_/C, which is in line with the very limited S-absorption performance of iron described in an earlier work^19^. In spent MO_x_/C (M = Zn, Cu, Mn, and Ce), crystalline sulfides are formed as ZnS, Cu_1.8_S, MnS, and Ce_2_S_3_, respectively, revealing the theoretical S-absorption capacity of those materials. Combining with the experimental S-absorption capacity presented in Fig. 3b, the sulfidation rate of those S-absorbents were evaluated and are listed in Table 3. In terms of S-absorption efficiency, CuO_x_/C shows much better performance than ZnO/C, MnO_x_/C, and CeO_x_/C. The material stability of those S-absorbents under such reaction conditions is another important aspect, which was evaluated by material characterization in the next section.

### Different structural evolution of MO_x_/C materials under HTG conditions

#### Evolution of ZnO/C by SCW and upon sulfidation under HTG condition

XRD and representative TEM and EDX results for as-prepared, SCW-treated, and sulfided ZnO/C are presented in Fig. 4.

##### The effect of SCW

As mentioned in “Characterization of the as-prepared MO_x_/C materials” section, the as-prepared ZnO/C (Fig. 4a) is composed of ZnO NPs with the particle size 14 ± 5 nm, homogeneously distributed in a carbon matrix (Fig. S5a). After exposure to SCW (water at 450 °C and 30 MPa) for 24 h, ZnO particles migrated outward from the C matrix along with a size increase of the particles as shown in Fig. 4b, in line with the increased crystallite size from 19 to 29 nm indicated by their respective XRD patterns (Fig. 4d). Little literature exists on the mechanisms of particle growth from a supported porous material under supercritical water conditions. The high surface energy and thermodynamic potential inherent to nanoparticles are a driving force for nanoparticle growth, and they are considerably worsened by supercritical water conditions. Coarsening typically occurs via Ostwald ripening, by migration of the particle and their coalescence, or by a combination of both phenomena^40^.

In the case of Ostwald ripening, particles would be immobile in the carbon matrix and monomers of zinc oxide would migrate from particles below a critical size to larger ones. This phenomenon can occur by the dissolution of monomers and/or via surface migration. The variation of ZnO solubility in water from standard conditions to SCW state (450 °C, 30 MPa, ρ_H2O_ = 0.144 g mL^−1^) can be one reason for such ripening and has been explored in various studies^41,42^. Thermodynamic modeling in the next “Thermodynamic modeling of MO_x_ dissolution in water” section will estimate the solubility of ZnO in water along the heating-up process from liquid water (50 °C, 3.5 MPa) to supercritical water (450 °C, 30 MPa). This variation of solubility upon heating (and cooling) under pressurized water might favor particle growth by Ostwald ripening.

Another coarsening mechanism is through the migration of particles and their subsequent coalescence. The majority of the work done on particle aggregation in supercritical water covers salt precipitation or particle synthesis, which are dominated by supersaturation of an ionic solution^43,44^. Small clusters with large charge imbalance were calculated by molecular dynamics to be readily formed and to dominate the growth of FeCl_2_ clusters^45^. Low Tammann point, i.e. close to the operation temperature, favors particle migration and coalescence, but in the case of ZnO the Tammann point is rather high (850 °C).

More specifically, several parameters influence particle migration, such as ionic strength, cation species or pH, but also the structural hydration layer thickness of nanoparticles that increases with decreasing particle size^46^. Under SCW conditions, it was shown that the thickness of the hydration layer increases with temperature, doubling from 400 to 500 °C, while an increase of pressure led to a decrease of that layer^46^. It can be considered that a hydration layer might be favored on zinc oxide under the reaction conditions used in this work.

In pure supercritical water, both Ostwald ripening and particle migration/coalescence might be involved in the growth of particle size. Many coarsened ZnO particles present a nearly defect-free rod-like morphology, indicating the growth of hexagonal ZnO crystallite in the direction [001].

##### Evolution upon sulfidation under HTG condition

The ZnO agglomerates and rods are also found in the spent ZnO/C after the S-absorption capacity test with DEDS (Fig. 4c), which caused the broadening of PSD throughout the material (Fig. S5a). ZnO was not fully sulfided because of a stoichiometric excess of ZnO in the feed (M/S = 2 mol_M_ mol_S_^−1^). The lattice space ZnO [100] of a sidewall of one rod was measured from an HR-TEM image (Fig. S8), which confirms the length-wise growth of hexagonal ZnO along its perpendicular direction [001]^47^. STEM-EDX elemental mapping indicates an irregular shape of ZnS particles with a size up to 400 nm and a representative Zn/S atom percentage to be roughly 50/40. More importantly, the distribution of S throughout the sample was very heterogeneous, with some Zn particles having an O:S ratio of 1, while other particles had an O:S close to 0 (see Fig. 4c). Overall, EDX data indicate that S combines with Zn by replacing O, confirming zinc oxide sulfidation, as expected from exposing this material to H_2_S (from DEDS decomposition^24^).

Trisulfide radical ion S_3_^⋅−^ was evidenced to be a ubiquitous sulfur species in reductive S-rich hydrothermal fluids, and has been shown to coordinate with Pt(II), Pt(IV), and Au(I) to form soluble and stable Pt^II^(HS)_2_(S_3_)_2_^2−^, Pt^IV^(HS)_3_(H_2_O)(S_3_)_2_^−^, and Au(HS)S_3_^−^ in hydrothermal fluids^48,49^. These complexes probably explain the migration of elements in the Earth's crust and the formation of ores rich in these metals. It is hypothesized that all chalcophile elements such as Zn(II) might form similar hydrated trisulfide complexes in reductive SCW^50^. Such complexes would hence favor particle growth of ZnS particles, e.g. by Ostwald ripening, but the absence of large ZnS crystals (Fig. 4d) and the presence of small ZnS particles aggregated in defined structure, e.g. rod-like (Fig. 4c), indicate that this mechanism is not favored. Similar to the conclusion from Tiemann et al.^51^ who modeled the kinetics of ZnS NPs growth at standard T&P, the mechanism of ZnS growth corresponds to coalescence with barrier-controlled attachment rather than Ostwald ripening. Indeed, Ostwald ripening involves material dissolution that is less favored for ZnS (K_sp_ = 10^–24^) than for ZnO (K_sp_ = 10^–16^ for Zn(OH)_2_)^52^.

Detailed STEM-EDX study of a sulfided rod-like particle (Fig. 4c, top right) indicates a particle with a rough surface composed of aggregated 3–5 nm ZnS NPs and two ~ 50 nm partially crystalline ZnO particles at the tip of the needle. First, this observation indicates that crystallization of ZnS particles are not favored under such conditions but that aggregation is strongly favored as no free 3–5 nm particles were observed. This is in line with the findings of Ma et al. who observed that sulfidation of the ZnO NPs decreases their surface charge and their Zeta potential, promoting their aggregation, compared to pristine ZnO NPs at standard pressure and temperature^52^. The structure of ZnO core covered by ZnS NPs shell resembles what Ma et al.^52^ found when sulfiding ZnO NPs in NaSH aqueous solution at room temperature and pressure. These observations would hence support the hypothesis that ZnS is formed by the dissolution of Zn oxide and its precipitation in the form of ZnS.

Based on the observations made and the existing literature, it can be hypothesized that during the S-absorption tests performed, ZnO coarsened probably via competitive Ostwald ripening (ZnO dissolution before ZnO monomers/nuclei migration) and particle migration/coalescence, yielding to crystalline needle-like particles and round poorly crystalline ones. In parallel to this, sulfidation of ZnO occurred likely by ZnO dissolution and then ZnS precipitation with hydrated zinc trisulfide complexes as the intermediates, yielding ZnS NPs agglomerated in place of bulk ZnO particles.

#### Evolution of CuO_x_/C by SCW and upon sulfidation under HTG condition

Figure 5 summarizes the XRD and representative TEM and EDX results for as-prepared, SCW-treated, and sulfided CuO_x_/C after the S-absorption capacity tests.

##### The effect of SCW

The as-prepared CuO_x_/C, as mentioned in “Characterization of the as-prepared MO_x_/C materials” section, consists of 26 ± 7 nm Cu_2_O/Cu NPs randomly distributed in the porous C matrix (Figs. 5a, S5b), the SCW-treated CuO_x_/C (see Fig. 5b) shows a distinct separation of porous C matrix and CuO_x_ particles with an increase of particle size. Two different morphologies of CuO_x_ particles are presented, which are hollow nanospheres (d = 40–70 nm) and micrometric agglomerates. The corresponding XRD pattern (red curve in Fig. 5d) indicates a decrease of crystalline Cu_2_O in favor of Cu(0) when comparing SCW-treated CuO_x_/C to the as-prepared material. SCW has no redox effect on Cu_2_O and Cu(0) as evidenced by Pocock et al.^53^. This change in diffraction pattern could be explained by the reduction of copper by organic compounds formed by exposing the carbon matrix to SCW. The difference in Tammann points of Cu_2_O and Cu(0) (480 °C and 406 °C, respectively) would lead Cu(0) to sinter more readily than Cu_2_O and would also favor the formation of larger crystals after SCW at 450 °C. The decreased crystallinity of Cu_2_O could be explained by the Ostwald ripening process with crystal dissolution as the onset, supported by its higher solubility in SCW than Cu(0)^53^ which will be also proved by thermodynamic modeling in the next “Thermodynamic modeling of MO_x_ dissolution in water” section. In line with the findings by Zhang et al., the hollowing process of Cu_2_O spherical particles was driven by Ostwald ripening mostly through aqueous copper ions migration and formed ultimately poorly crystalline Cu_2_O hollow spheres^54,55^. Based on the literature and the XRD result (red curve in Fig. 5d), a plausible explanation to morphology evolution of CuO_x_/C under SCW is that the hollow nanospheres are Cu_2_O nanocrystallites formed by Ostwald ripening, while the micrometric agglomerates are formed by migration/coalescence of Cu(0) and Cu_2_O.

##### Evolution upon sulfidation under HTG condition

After reacting with DEDS in the S-absorption capacity test with M/S = 2 mol_M_ mol_S_^−1^, the crystalline constituents of spent CuO_x_/C are Cu_2_O (average D_p_ = 19 nm) and Cu_1.8_S (average D_p_ = 31 nm) as indicated by XRD (green curve in Fig. 5d). The maintained peak intensity of Cu_2_O and the absence of Cu(0), as opposed to the SCW-treated CuO_x_/C, suggests a preferential sulfidation of Cu(0) rather than Cu_2_O. This is in line with the work of Ziegler et al.^56^ who suggested that in the presence of hexanethiol in SCW environment, CuO firstly reduced to Cu(0) on which thiol adsorbs. Ethanethiol being an intermediate decomposition product from DEDS in SCW (cf. Fig. S6b), one would expect it to be adsorbed to Cu(0) surface before further decomposing to Cu_x_S and ethylene. In this work, Cu_2_O is more likely reduced to Cu(0) by isopropanol or its decomposition products. STEM-EDX study of the spent material indicates a very heterogeneous sulfidation of CuO_x_/C as observed with ZnO/C (see Fig. 5c). EDX results indicate that Cu- and S-rich particles are generally shapeless and often reach a size of a few hundred nanometers. The semi-quantitative EDX analysis of S-rich Cu particles suggests an atomic proportion of Cu, S, and O to be 65%, 18%, and 1%, respectively, indicating roughly a S/Cu ratio of 0.28 mol_S_ mol_Cu_^−1^ in the selected region. Considering the theoretical S-absorption capacity of 0.56 mol_S_ mol_Cu_^−1^ for Cu_1.8_S observed by XRD, this area shows a 50% sulfidation rate, higher than the 31% of the bulk material (Table 3) due to the presence of non-sulfided copper. The EDX analysis of a Cu-rich but S-free area indicated 32 mol%, 1 mol%, and 15 mol% of Cu, S, and O, respectively, in line with Cu_2_O. Hollow Cu_2_O particles with a diameter ranging from 30 to 70 nm are also found, like those observed on the SCW-treated CuO_x_/C in Fig. 5b.

Exposing CuO_x_/C to an equimolar amount of sulfur (M/S = 1) with a longer HTG reaction time (15 h) enabled Cu_1.8_S (D_p_ = 34 nm) to be the predominant crystalline phase in the sample as outlined by XRD (purple curve in Fig. 5d). The Cu_2_O pattern is nearly invisible, and Cu(0) inexistent, indicating an extensive sulfidation of copper, in line with the high sulfidation rate (77%, see Table 3) in the spent material. After this long sulfidation test, TEM indicated a material composed of 20–100 nm NPs (majorly Cu_1.8_S) distributed within/on the C matrix, but also some large particle/agglomerate of a few hundred nanometers (see Fig. S9). Well-distributed < 5 nm nanocrystallites were also observed in the sample (Fig. S10), expected to be Cu_1.8_S and Cu_2_S according to lattice analysis. Considering their small size and low abundance, it is possible that these particles (at least the Cu_2_S particles) formed from a rapid reaction between dissolved Cu(H_2_O)_x_^+^ and HS^−^ upon quenching the reactor, a reaction that was shown to occur within a few minutes at room temperature^57^.

Overall, the results indicate a preferential sulfidation of Cu(0) over Cu_2_O, and the partial reduction of Cu(I) to Cu(0). Cu(0) sulfidation likely occurs by surface adsorption of H_2_S, or thiol followed by its subsequent decomposition and subsequent migration of S within already partially sintered Cu particles. Cu_2_O sulfidation seems to follow a dissolution/precipitation mechanism as suggested for ZnO.

#### S-inert FeO_x_/C with high structural stability under HTG condition

FeO_x_/C presents high stability upon hydrothermal treatment with DEDS. No iron sulfide compounds could be detected by XRD. This is in contradiction with the absorption capacity results, indicating that the carbon matrix absorbed more S than expected from the absorption observed on the reference C sample. Comparing TEM (Fig. 6a,b) and PSD (Fig. S5c) results of spent and fresh FeO_x_/C, a limited impact of SCW and DEDS/isopropanol on the material (see Fig. 6c) was found, with a limited coarsening of FeO_x_ particles growing from 15 ± 4 to 18 ± 5 nm, in phase with the limited crystallite growth observed by XRD (D_p_ from 20 to 36 nm in average). Overall, the particle size distribution remains narrow and the particles are homogeneously distributed within the C matrix. The only crystalline phase in the fresh material and the material after the sulfidation test was Fe_3_O_4_, but in the latter, very weak diffraction peaks corresponding to Fe_2_O_3_ were observed.

**Figure ﻿6:**
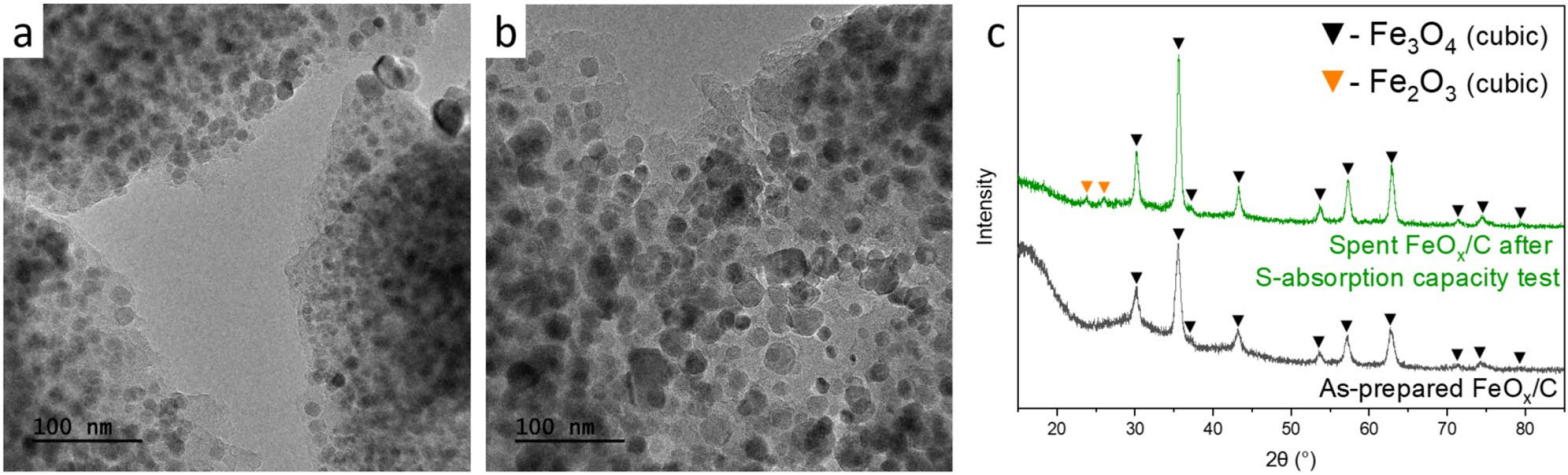
TEM images of (**a**) as-prepared FeO_x_/C, (**b**) spent FeO_x_/C after S-absorption capacity test with DEDS model feed. (**c**) XRD patterns of these two materials.

These observations further confirm the poor ability of iron oxide to absorb sulfur. This is due to the lack of stability of iron sulfide in supercritical water previously reported^19,58^. This finding also discloses the high stability of the oxide with limited coarsening/sintering in SCW conditions. This property makes FeO_x_/C or Fe_3_O_4_ suitable materials for other applications under SCW conditions, such as catalyst support or guard bed for downstream heterogeneous catalysts to retain e.g. transition metals or particles.

#### Evolution of MnO_x_/C upon sulfidation under HTG conditions

MnO_x_/C suffered from particle agglomeration, similar to ZnO/C and CuO_x_/C, after reacting with DEDS in the SCW environment. Big agglomerates are ubiquitous in the spent material as shown in Fig. 7b, with heterogeneous distribution of sulfur. The formation of MnS was confirmed by XRD shown in Fig. 7c. MnO is the major crystalline phase in fresh MnO_x_/C and after exposure to sulfur under SCW. The mean crystallite size D_p_ decreased from 45 to 38 nm. The EDX results of a representative S-rich area suggests Mn, S, and O concentrations to be 26 mol%, 11 mol%, and 14 mol%, respectively. The sulfidation ratio of this area reaches 42%, showing a big difference compared to the bulk ratio of 9.3% (see Table 3). The heterogeneity of S-absorption has been also observed on porous ZnO/C and CuO_x_/C. It seems that the porosity of the MO_x_/C material has a very limited impact on the S-absorption homogeneity.

#### CeO_x_/C with high structural stability upon sulfidation under HTG conditions

After the S-absorption reaction with DEDS under hydrothermal conditions, no large agglomerates are found in the spent CeO_x_/C, but some growth of the NPs from below 2–3 nm (Fig. 8a) to 5 ± 2 nm (Fig. 8b). The particle size distribution (see Fig. S5e) remains narrow and the particles are still homogeneously distributed within the C matrix. The average crystallite size of the main component CeO_2_ increased from 4.1 to 5.7 nm with an increase of crystallinity, as indicated by XRD (Fig. 8d). Another very weak pattern is tentatively assigned to Ce_2_S_3_. EDX in Fig. 8c indicates a rather homogeneous distribution of S throughout the material, with a spatial distribution closer to that of C and O than Ce. A small proportion of S-rich areas are also rich in Ce, e.g. the top-left region, which should correspond to small highly-sulfided CeS_x_ NPs.

In summary of “Different structural evolution of MO_x_/C materials under HTG conditions” section, the above material characterizations disclose the significant morphological evolution of ZnO/C, CuO_x_/C, and MnO_x_/C by reacting with DEDS under HTG conditions. The distinct separation of MO_x_ (M = Cu, Zn, Mn) from porous C matrix and the formation of big M-based agglomerates were observed. In contrast, FeO_x_/C and CeO_x_/C showed outstanding structural stability with MO_x_ particle size remaining well-dispersed within the C matrix and below 36 and 6 nm, respectively. FeO_x_/C presented a limited capacity to absorb sulfur with no crystalline Fe sulfides generated. CeO_x_/C showed a good S-absorption capacity comparable to ZnO/C, but lower than CuO_x_/C, with the formation of small cerium sulfide NPs. S-absorption on the non-porous CeO_x_/C were homogeneous, in contrast with the porous ZnO/C and CuO_x_/C, which however showed heterogeneous S-absorption. It seems that the porosity of the MO_x_/C material has a very limited impact on the S-absorption homogeneity. A bold assumption is a faster rate of MO_x_/C structural evolution (MO_x_ particle size growth and their migration away from the C matrix) than S-absorption under such conditions.

Ostwald ripening, initiated by the dissolution of MO_x_ particles, might be responsible for the migration of the Zn, Cu and Mn oxides away from the C matrix and the particle growth. In the next section, we use thermodynamic models to predict the dissolution of ZnO, Cu_2_O, Cu, Fe_3_O_4_, MnO, and CeO_2_ in water under the conditions (*T, P, ρ*_*H2O*_) applied in the experimental part of this paper to give further support to this assumption.

### Thermodynamic modeling of MO_x_ dissolution in water at different conditions

**Figure 9:**
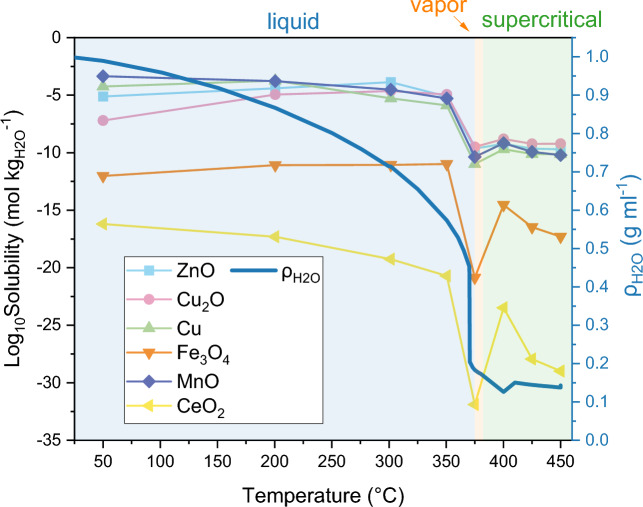
Calculated solubility of ZnO, Cu_2_O, Cu, Fe_3_O_4_, MnO, and CeO_2_ in pure neutral water at eight different sets of conditions (*T*, *P*, *ρ*_*H2O*_) along the heating-up process from liquid water (50 °C, 3.5 MPa, 0.99 g mL^−1^) to supercritical state (450 °C, 30 MPa, 0.14 g mL^−1^) during a batch reactor test.

Reported solubility data of ZnO, Cu_2_O, Cu, Fe_3_O_4_, MnO, and CeO_2_ under sub/supercritical water conditions are scarce in the literature. Based on the R-HKF method illustrated in “Thermodynamic modeling of MO_x_ dissolution in water”, the solubility of those metal oxides or metals in pure neutral water was calculated for eight different sets of conditions (*T, P, ρ*_*H2O*_) along the heating-up process from liquid water (50 °C, 3.5 MPa, 0.99 g mL^−1^) to supercritical water (450 °C, 30 MPa, 0.14 g mL^−1^). The calculated solubility result is tabulated in Table S7 and shown in Fig. 9. Shock et al.^30^ calculated ZnO solubility in 200 °C pure neutral water as 10^–4.4^ mol kg^−1^, the same as the calculated value in this paper. Bénézeth et al.^59^ measured ZnO solubility in water containing a trace amount of NaOH (350 °C, 17.5 MPa, pH = 8.174) as 10^–5.6^ mol kg^−1^, with a very small difference to the value calculated in this paper, 10^–5.1^ mol kg^−1^, for similar *T* and *P*.

For all of those MO_x_ materials, a higher solubility in hot liquid water (≤ 350 °C) than in supercritical water (≥ 400 °C) is observed (Fig. 9), while the lowest solubility is found at conditions where water is majorly in the vapor state (point at 375 °C and 217 bar) due to a rapid decrease of water’s dielectric constant. Figure 9 indicates very limited solubility of Fe_3_O_4_ and CeO_2_ even in hot liquid water, with values lower than 10^–10^ mol kg^−1^ and 10^–16^ mol kg^−1^, respectively. The solubility of ZnO, Cu_2_O, Cu, and MnO were found to be close to each other, and was calculated to be over five orders of magnitude higher than Fe_3_O_4_ and nine orders of magnitude higher than CeO_2_. ZnO and Cu_2_O both show a dissolution increasing with a temperature rising from 50 to 300 °C, reaching at 300 °C their highest solubility of 10^–3.9^ mol kg^−1^ and 10^–4.6^ mol kg^−1^, respectively. MnO is predicted to be preferentially dissolved in colder water and it reaches its highest solubility of 10^–3.4^ mol kg^−1^ at 50 °C. The highest solubility of Cu(0) at the modeled conditions is predicted to be 10^–3.7^ mol kg^−1^ at 200 °C.

These predictions agree with the experimental evidence of the structural evolution of MO_x_/C discussed in “Different structural evolution of MO_x_/C materials under HTG conditions” section. FeO_x_/C and CeO_x_/C showed outstanding structural stability with their MO_x_ (M = Fe, Ce) NPs still well-dispersed within the C matrix after the HTG reaction, in line with the low solubility of Fe_3_O_4_ and CeO_2_ in water. In contrast, the migration of MO_x_ (M = Zn, Cu, Mn) particles from the C matrix along with the growth of particle size observed on spent MO_x_/C (M = Zn, Cu, Mn) materials after HTG reaction correlate with the higher calculated solubility of ZnO, Cu/Cu_2_O, and MnO in sub/supercritical water. TEM analysis of spent materials brought evidence that Ostwald ripening is involved in the particle growth at least of Zn- and Cu-based particles (“Evolution of ZnO/C by SCW and upon sulfidation under HTG condition” and “Evolution of CuO_x_/C by SCW and upon sulfidation under HTG condition” sections). The correlation between higher particle growth and higher solubility supports the occurrence of Ostwald ripening. When it comes to tests in presence of a large amount of reduced sulfur compounds, the model used here does not apply anymore. Indeed, the acidification effect of dissolved H_2_S (decrease of pH) might promote the dissolution of MO_x_ by a few orders of magnitude^59^. The correlation between the M-based particle growth and the heterogeneity of S-absorption was also observed by EDX. This suggests that MO_x_ with different solubility have different S-absorption mechanisms. MO_x_ with relatively high solubility, such as ZnO, Cu_2_O, and MnO, may absorb S by dissolution (to metal cations) and reprecipitation (forming metal sulfide species with lower solubility). In contrast, sulfidation of very low solubillity CeO_2_ NPs may occur through surface adsorption of sulfur species (likely H_2_S or thiol) followed by S migration in the bulk of the material leading to the formation of cerium sulfide.

## Conclusions

Metal oxide nanoparticles embedded in carbon, MO_x_/C (M = Cu, Ce, Zn, and Mn), have been shown to absorb sulfur at HTG conditions with different S-absorption capacities with the following order CuO_x_/C > CeO_x_/C ≈ ZnO/C > MnO_x_/C, through the formation of Cu_1.8_S, Ce_2_S_3_, ZnS, and MnS nanocrystals, respectively. However, the lack of stability observed for some active phases, such as Cu_2_O/Cu, ZnO, and MnO, could lead to a non-negligible loss of performance and mechanical loss of material in a continuous flow process. Calculations showed that the solubility of the active phases is low, and thus they would not leach significantly during a continuous hydrothermal processing of wet biomass under supercritical water conditions. A correlation between MO_x_ solubility and MO_x_/C structural evolution observed in the spent material, i.e. particle growth/migration of particle outside the C matrix, was found. Together with the evolution of particle size distribution, this indicates that Ostwald ripening through metal dissolution is likely the dominating cause for particle size growth. Interestingly, coalescence seems to be less favorable with sulfided metals than with the corresponding metal oxides, which is particularly visible for ZnO. Consequently, sulfidation of relatively more soluble metal oxides under HTG condition is believed to follow MO_x_ dissolution/MS_x_ precipitation mechanism, with strong evidence for ZnO, Cu_2_O, and MnO. The kinetics of nuclei migration in SCW, and the stability of the formed metal sulfide particles in long-term operation remains to be determined.

Fe_3_O_4_ was found to have a high stability with very low capacity to absorb sulfides during the HTG process. These properties make this material an excellent candidate as a long-lasting safeguard bed to protect downstream catalysts from poisoning by other transition metals or solids, or as a stable support in composite S-absorbents. CeO_x_/C exhibited a high structural stability and good S-absorption capacity, and is suggested as a promising sulfur absorbent in such applications. Further efforts should be devoted towards developing the synthesis of CeO_x_/C to achieve high CeO_x_ loading with high porosity, as well as efforts in assessing the regeneration of the sulfided material.

## Supplementary Information

Supplementary Information.

## Data Availability

The datasets generated and/or analysed during the current study are available from the corresponding author on reasonable request.
