# Supplementary material for: Metal oxide nanoparticles embedded in porous carbon for sulfur absorption under hydrothermal conditions
Source: Sci Rep. 2023 Jun 20;13:9987. doi: 10.1038/s41598-023-36395-8 (PMC10282018; doi:10.1038/s41598-023-36395-8)
Supplement: Supplementary file 1 — Supplementary Information. [file 41598_2023_36395_MOESM1_ESM.docx]

Supplementary Information

Metal oxide nanoparticles embedded in porous carbon for sulfur absorption under hydrothermal conditions

Authors

Hang Xiang^1^, David Baudouin^1^^[[1]](#footnote-1)^, Frédéric Vogel^1,2^.

Author Information

^1^ Laboratory for Bioenergy and Catalysis, Paul Scherrer Institute (PSI), 5232 Villigen PSI, Switzerland

^2^ University of Applied Sciences Northwestern Switzerland (FHNW), 5210 Windisch, Switzerland

Keywords

Desulfurization; Sulfur absorption; Supercritical water; Hydrothermal gasification; Metal carbon catalyst;


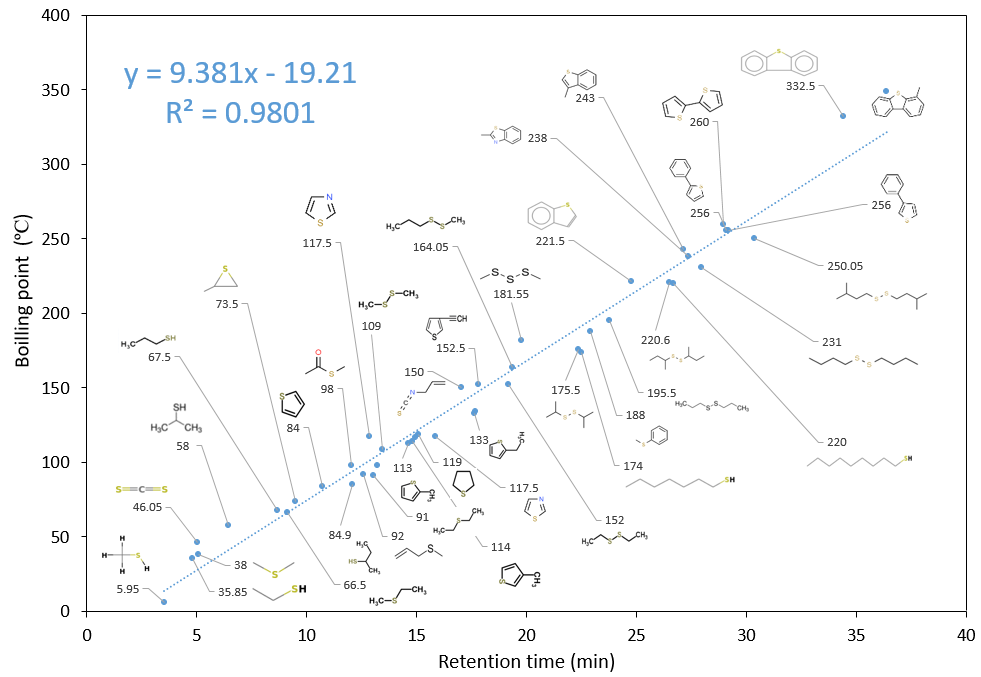


Figure S1 - The linear relationship between the boiling point (ºC) of the sulfur compounds and their retention time (min) determined by GC-SCD based on specific testing conditions


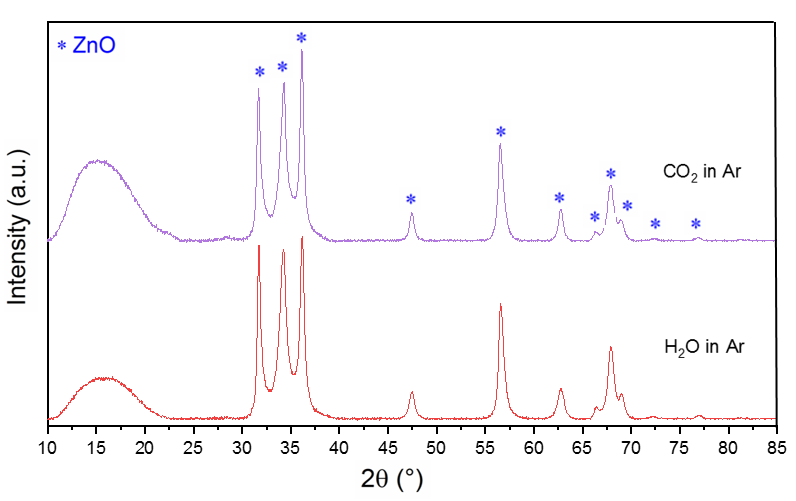


***Figure S2 - XRD patterns of as-prepared ZnO/C materials synthesized with different carrier gases in pyrolysis process: “1.4 vol.% H_2_O in Ar” and “1 vol.% CO_2_ in Ar”.***


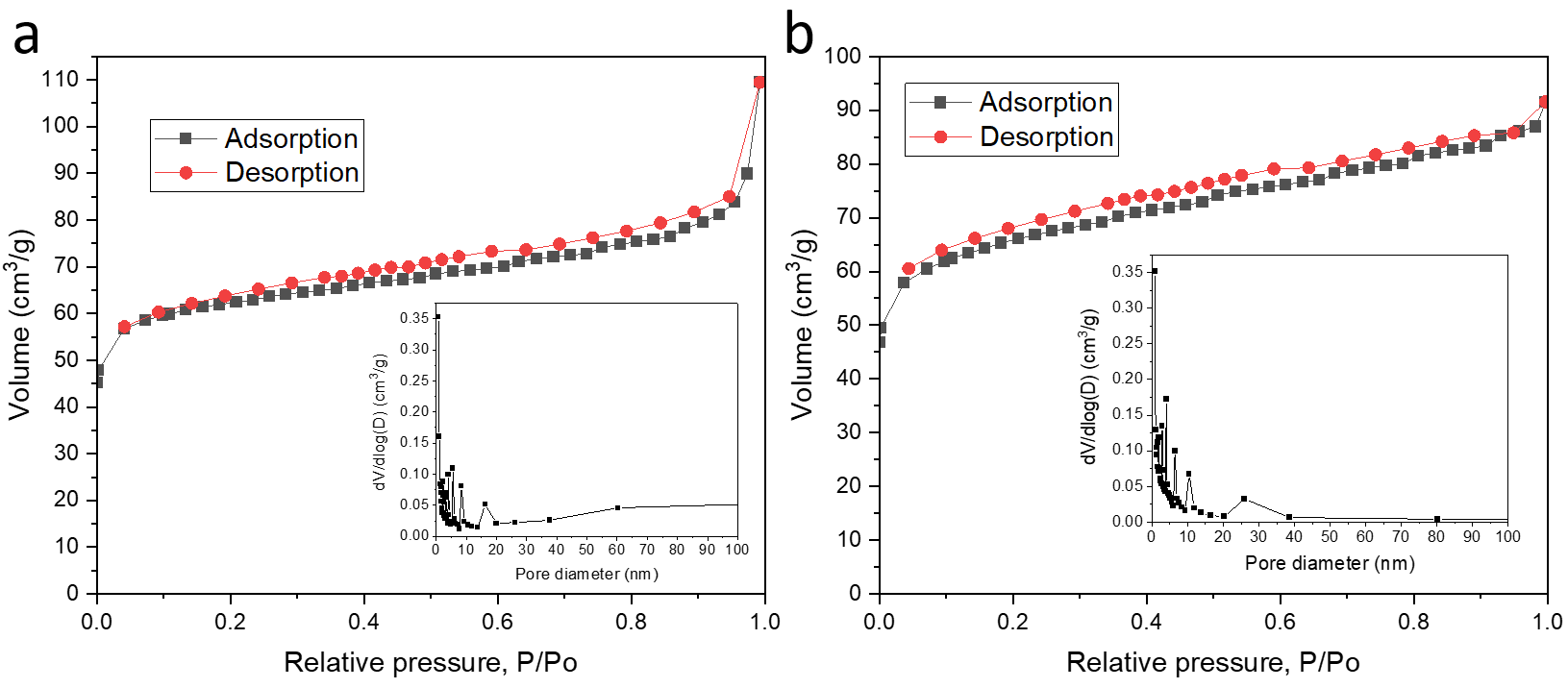

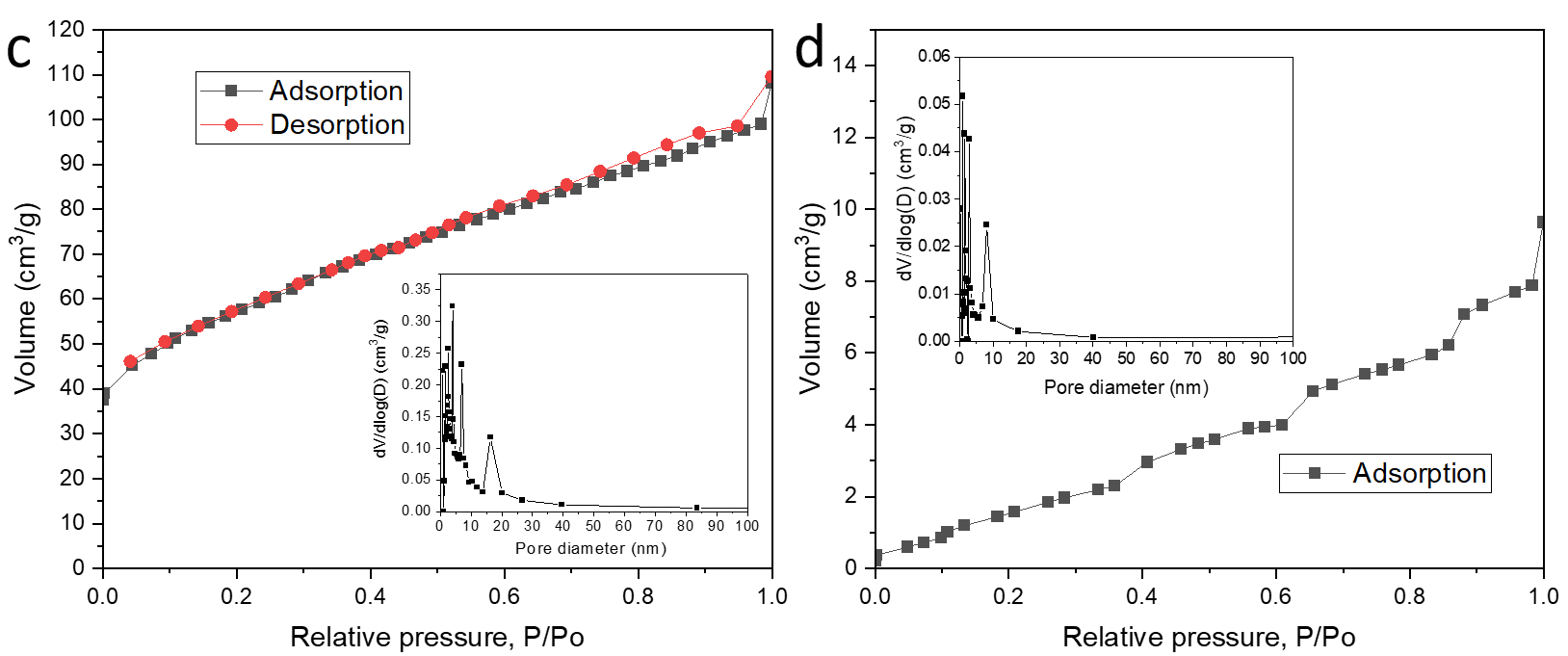


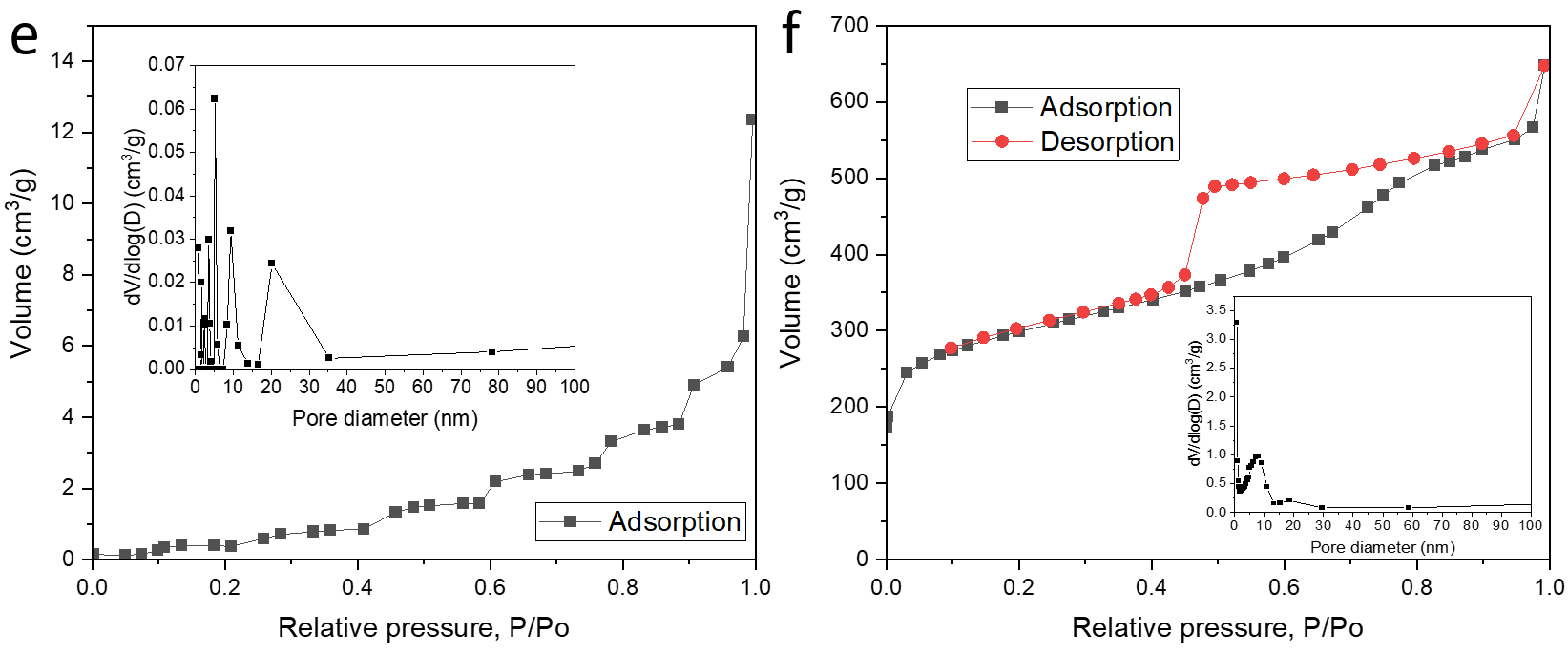


Figure S3 - N_2_ adsorption–desorption isothermal curves and pore size distribution of as-prepared a) ZnO/C, b) CuO_x_/C, c) FeO_x_/C, d) MnO_x_/C, e) CeO_x_/C, and f) C


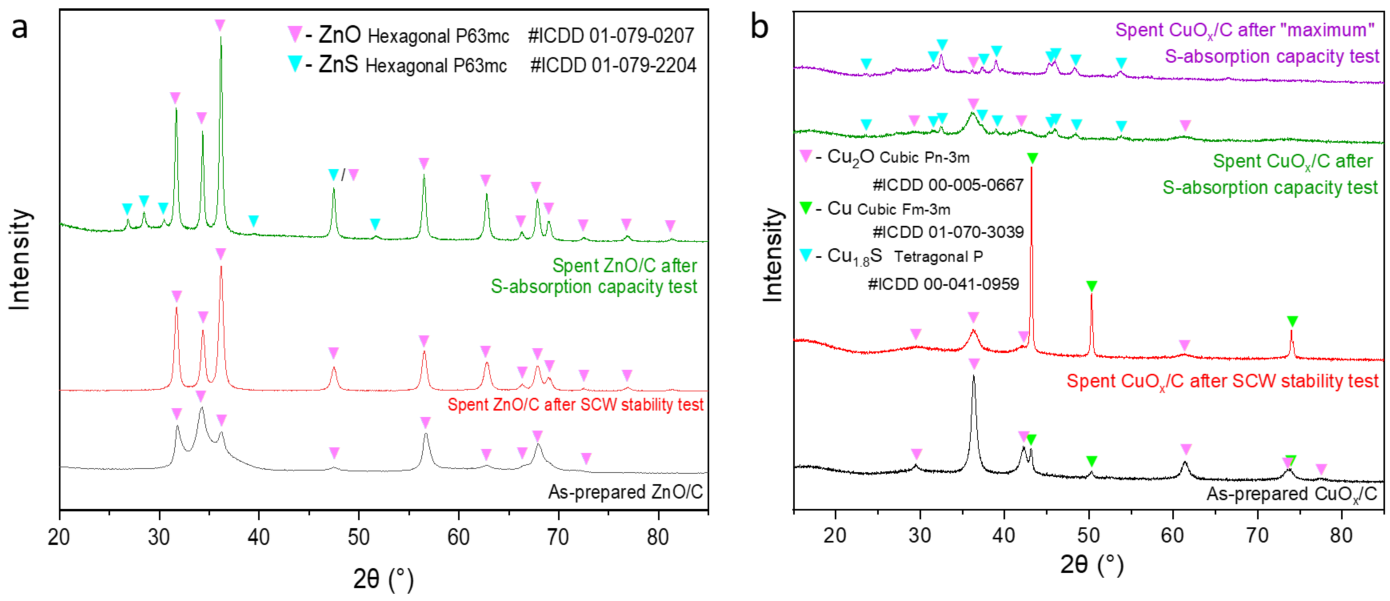


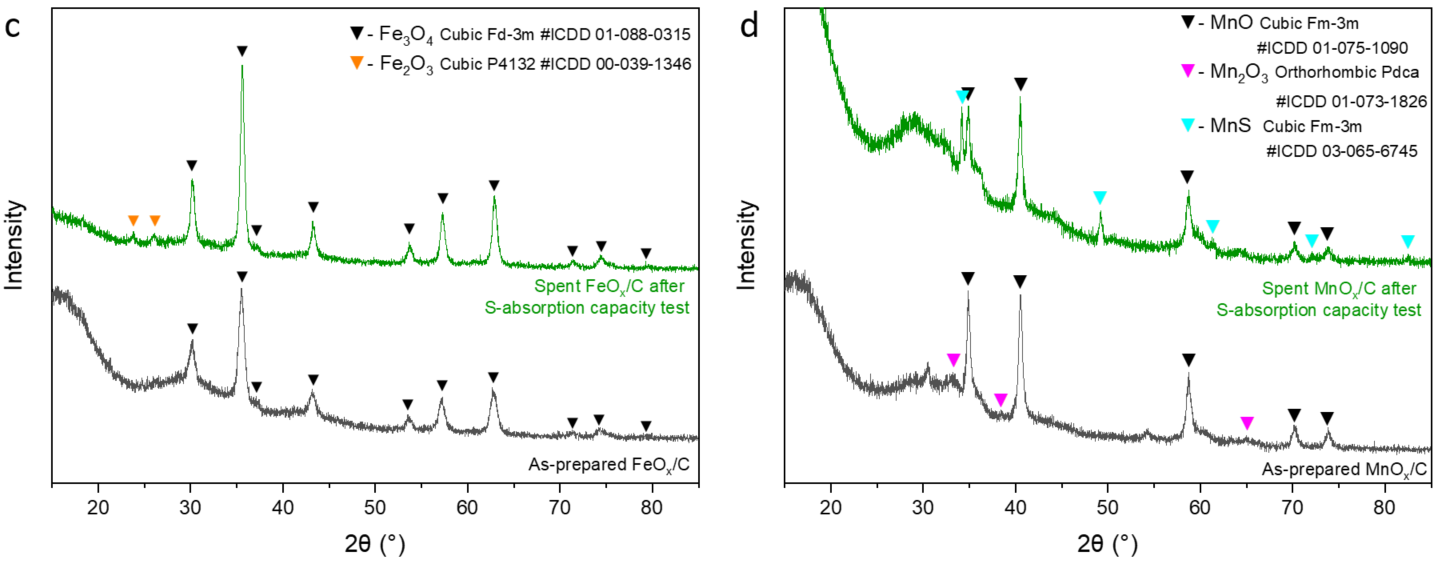


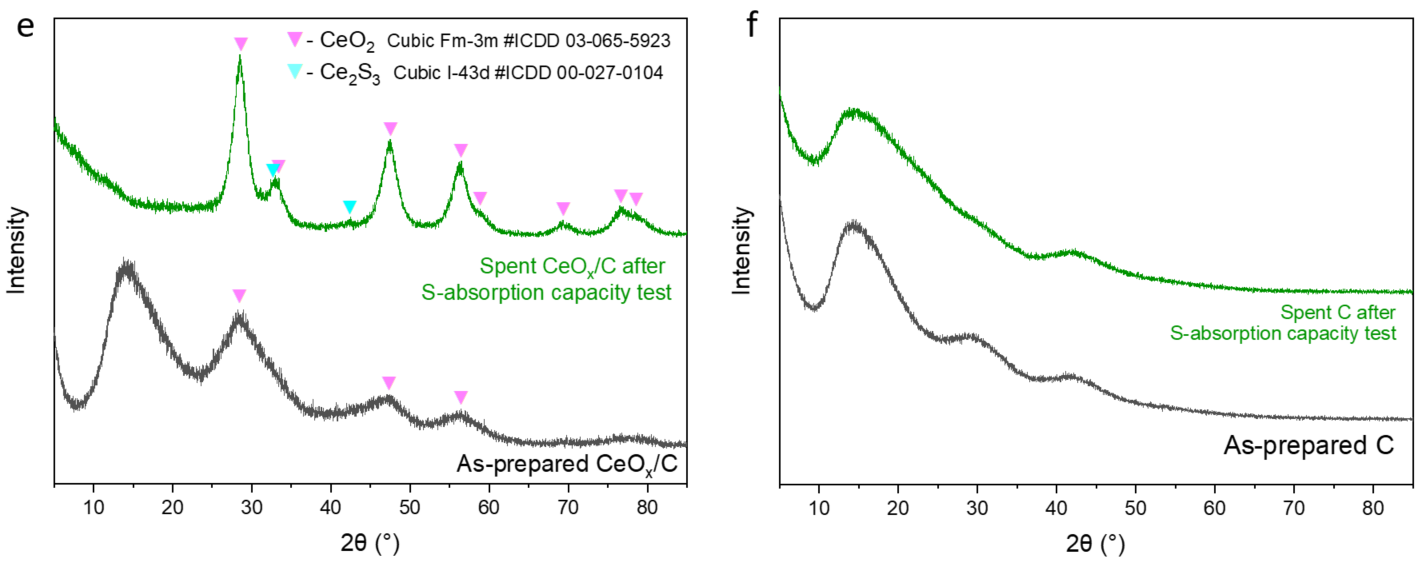


Figure S4 – XRD patterns of as-prepared and spent a) ZnO/C, b) CuO_x_/C, c) FeO_x_/C, d) MnO_x_/C, e) CeO_x_/C, and f) C. The pattern drawn in black, red, green, and purple respectively representes as-prepared materials, spent materials after “SCW stability” test (only ZnO/C and CuO_x_/C tested), spent materials after S-absorption capacity test, and spent materials after “maximum” S-absorption capacity” test (only CuO_x_/C tested).

Table S1 – Calculation of mean crystalline size of fresh and spent a) ZnO/C, b) CuO_x_/C, c) FeO_x_/C, d) MnO_x_/C, and e) CeO_x_/C using Scherrer Equation. Data was extracted from the XRD patterns in Figure S4.

| a) | **miller index** | **2θ (º)** | **As-prepared ZnO/C** | | **ZnO/C after SCW- stability test** | | **ZnO/C after S-** **absorption capacity test** | |
| --- | --- | --- | --- | --- | --- | --- | --- | --- |
|  |  |  | **B (º)** | **D_p_ (nm)** | **B (º)** | **D_p_ (nm)** | **B (º)** | **D_p_ (nm)** |
| **ZnO** Hexagonal P63mc  # ICDD 01-079-0207 | 1,0,0 | 31.81 | 0.30 | 28.0 | 0.21 | 39.2 | 0.22 | 37.5 |
|  | 0,0,2 | 34.24 | 0.64 | 13.0 | 0.29 | 28.6 | 0.19 | 44.0 |
|  | 1,0,1 | 36.20 | 0.39 | 21.2 | 0.28 | 29.4 | 0.24 | 35.4 |
|  | 1,0,2 | 47.51 | 0.69 | 12.6 | 0.31 | 28.1 | 0.13 | 68.9 |
|  | 1,1,0 | 56.70 | 0.44 | 20.4 | 0.39 | 22.9 | 0.15 | 58.7 |
|  | 1,0,3 | 62.81 | 0.59 | 15.8 | 0.29 | 32.2 | 0.16 | 59.9 |
|  | 1,1,2 | 67.87 | 0.46 | 20.7 | 0.36 | 26.3 | 0.17 | 55.4 |
|  | 2,0,1 | 68.99 | N.D.^a^ | -- | N.D. | -- | 0.31 | 31.4 |
|  | **Average** | |  | **18.8** |  | **29.5** |  | **48.9** |
| **ZnS** Hexagonal P3m1  #ICDD 01-089-2145 | 0,0,15 | 19.36 |  |  |  |  |  |  |
|  | 0,0,16 | 20.66 |  |  |  |  |  |  |
|  | 0,0,17 | 21.97 |  |  |  |  |  |  |
|  | 1,0,0 | 26.91 |  |  |  |  |  |  |
|  | 0,1,6 | 28.03 |  |  |  |  |  |  |
|  | 0,1,8 | 28.88 |  |  |  |  |  |  |
|  | 1,1,0 | 47.53 |  |  |  |  |  |  |
|  | 0,1,30 | 48.27 |  |  |  |  |  |  |
|  | 2,0,12 | 57.94 |  |  |  |  |  |  |
|  | **Average** | |  |  |  |  |  |  |
| **ZnS** Hexagonal P63mc #ICDD-01-079-2204 | 1,0,0 | 26.91 |  |  |  |  | 0.16 | 51.9 |
|  | 0,0,2 | 28.49 |  |  |  |  | 0.08 | 104.1 |
|  | 1,0,1 | 30.52 |  |  |  |  | 0.25 | 32.7 |
|  | 1,0,2 | 39.59 |  |  |  |  | 0.38 | 22.3 |
|  | 1,0,3 | 51.74 |  |  |  |  | 0.25 | 35.0 |
|  | **Average** | |  |  |  |  |  | **49.2** |

| b) | **miller index** | **2θ (º)** | **As-prepared CuO_x_/C** | | **CuO_x_/C after SCW-stability test** | | **CuO_x_/C after S-absorption capacity test** | | **CuO_x_/C after "maximum” S-** **absorption capacity test** | |
| --- | --- | --- | --- | --- | --- | --- | --- | --- | --- | --- |
|  |  |  | **B (º)** | **D_p_ (nm)** | **B (º)** | **D_p_ (nm)** | **B (º)** | **D_p_ (nm)** | **B (º)** | **D_p_ (nm)** |
| **Cu_2_O** Cubic Pn-3m #ICDD 00-005-0667 | 1,1,0 | 29.56 | 0.31 | 26.1 | 1.51 | 5.4 | N/A | -- | N.D. | -- |
|  | 1,1,1 | 36.42 | 0.10 | 87.1 | 0.57 | 14.7 | 0.31 | 26.5 | 0.25 | 33.2 |
|  | 2,0,0 | 42.30 | 0.19 | 44.3 | 0.63 | 13.5 | N/A | -- | N.D. | -- |
|  | 2,2,0 | 61.35 | 0.19 | 48.1 | 1.13 | 8.1 | 0.77 | 12.0 | N.D. | -- |
|  | 3,1,1 | 73.53 | 0.31 | 32.3 | N.D. | -- | N.D. | -- | N.D. | -- |
|  | 2,2,2 | 77.33 | 0.23 | 44.1 | N.D. | -- | N.D. | -- | N.D. | -- |
|  | **Average** | |  | **47.0** |  | **10.5** |  | **19.3** |  | **33.2** |
| **Cu** Cubic Fm-3m #ICDD 01-070-3039 | 1,1,1 | 43.19 | 0.10 | 89.0 | 0.09 | 90.4 |  |  |  |  |
|  | 2,0,0 | 50.30 | 0.23 | 38.1 | 0.11 | 79.6 |  |  |  |  |
|  | 2,2,0 | 73.89 | 0.31 | 32.3 | 0.10 | 103.5 |  |  |  |  |
|  | **Average** | |  | **53.1** |  | **91.2** |  |  |  |  |
| **Cu_1.8_S** Tetragonal P  #ICDD 00-041-0959 | 2,0,0 | 23.50 |  |  |  |  | 0.38 | 21.5 | 0.38 | 21.5 |
|  | 1,0,5 | 27.25 |  |  |  |  | 0.38 | 21.6 | 0.16 | 51.9 |
|  | 0,1,6 | 31.56 |  |  |  |  | 0.44 | 18.7 | 0.31 | 26.2 |
|  | 1,2,4 | 32.52 |  |  |  |  | 0.13 | 65.7 | 0.25 | 32.8 |
|  | 1,3,0 | 37.51 |  |  |  |  | 0.22 | 38.1 | 0.13 | 66.6 |
|  | 1,3,2 | 39.03 |  |  |  |  | 0.19 | 44.6 | 0.28 | 29.7 |
|  | 1,2,6 | 39.77 |  |  |  |  | N.D. | **--** | 0.25 | 33.5 |
|  | 1,3,5 | 45.35 |  |  |  |  | 0.31 | 27.3 | 0.22 | 39.0 |
|  | 0,1,9 | 46.09 |  |  |  |  | 0.23 | 37.5 | 0.22 | 39.2 |
|  | 2,2,7 | 48.54 |  |  |  |  | 0.54 | 16.2 | 0.13 | 69.1 |
|  | 3,3,1 | 51.56 |  |  |  |  | N.D. | **--** | 0.76 | 11.7 |
|  | 1,4,4 | 53.75 |  |  |  |  | 0.61 | 14.5 | 0.38 | 23.6 |
|  | 2,5,0 | 66.62 |  |  |  |  | N.D. | **--** | 0.44 | 21.6 |
|  | 4,4,1 | 70.78 |  |  |  |  | N.D. | **--** | 0.63 | 15.5 |
|  | **Average** | |  |  |  |  |  | **30.6** |  | **34.4** |

| c) | **miller index** | **2θ (º)** | **As-prepared FeO_x_/C** | | **FeO_x_/C after S-absorption capacity test** | |
| --- | --- | --- | --- | --- | --- | --- |
|  |  |  | **B (º)** | **D_p_ (nm)** | **B (º)** | **D_p_ (nm)** |
| **Fe_3_O_4_** Cubic Fd-3m  #ICDD 01-088-0315 | 2,2,0 | 30.16 | 0.38 | 21.8 | 0.19 | 42.6 |
|  | 3,1,1 | 35.52 | 0.38 | 22.1 | 0.28 | 29.0 |
|  | 2,2,2 | 37.16 | 0.25 | 33.3 | 0.41 | 20.4 |
|  | 4,0,0 | 43.17 | 0.44 | 19.4 | 0.19 | 44.4 |
|  | 4,2,2 | 53.56 | 0.44 | 20.2 | 0.16 | 54.3 |
|  | 5,1,1 | 57.10 | 0.57 | 16.0 | 0.16 | 56.5 |
|  | 4,4,0 | 62.70 | 0.50 | 18.5 | 0.28 | 31.9 |
|  | 6,2,0 | 71.14 | 0.76 | 12.9 | 0.31 | 29.5 |
|  | 5,3,3 | 74.19 | 0.76 | 13.2 | 0.38 | 25.8 |
|  | 4,4,4 | 79.17 | 0.50 | 20.5 | 0.25 | 39.5 |
|  | **Average** | |  | **19.8** |  | **35.8** |
| **Fe_2_O_3_** Cubic P1432 #ICDD 00-039-1346 | 1,1,0 | 15.00 |  |  | N.D. | -- |
|  | 2,1,0 | 23.82 |  |  | 0.25 | 32.2 |
|  | 2,1,1 | 26.13 |  |  | 0.25 | 32.4 |
|  | **Average** | |  |  |  | **32.3** |

| d) | **miller index** | **2θ (º)** | **As-prepared MnO_x_/C** | | **MnO_x_/C after S-** **absorption capacity test** | |
| --- | --- | --- | --- | --- | --- | --- |
|  |  |  | **B (º)** | **D_p_ (nm)** | **B (º)** | **D_p_ (nm)** |
| **MnO** Cubic Fm-3m  #ICDD 01-075-1090 | 1,1,1 | 34.93 | 0.19 | 44.1 | 0.25 | 33.0 |
|  | 2,0,0 | 40.55 | 0.38 | 22.4 | 0.28 | 29.9 |
|  | 2,2,0 | 58.69 | 0.13 | 72.3 | 0.19 | 48.2 |
|  | 3,1,1 | 70.15 | 0.44 | 22.0 | 0.38 | 25.7 |
|  | 2,2,2 | 73.77 | 0.16 | 63.1 | 0.19 | 52.6 |
|  | **Average** | |  | **44.8** |  | **37.9** |
| **Mn_2_O_3_** Orthorhombic Pdca #ICDD 01-073-1826 | 2,2,2 | 33.31 | 0.50 | 16.5 |  |  |
|  | 6,2,2 | 65.09 | 0.13 | 74.8 |  |  |
|  | **Average** | |  | **45.6** |  |  |
| **Mn_3_O_4_**  Tetragonal I4/amd #ICDD 00-001-1127 | 1,1,2 | 28.97 |  |  |  |  |
|  | 1,0,3 | 32.53 |  |  |  |  |
|  | 3,1,4 | 64.68 |  |  |  |  |
|  | **Average** | |  |  |  |  |
| **MnS** Cubic Fm-3m #ICDD 03-065-6745 | 2,0,0 | 34.26 |  |  | 0.16 | 52.8 |
|  | 2,2,0 | 49.24 |  |  | 0.13 | 69.3 |
|  | 2,2,2 | 61.36 |  |  | 0.25 | 36.7 |
|  | 4,0,0 | 72.19 |  |  | 0.38 | 26.0 |
|  | 4,2,0 | 82.40 |  |  | 0.25 | 41.9 |
|  | **Average** | |  |  |  | **45.3** |

| e) | **miller index** | **2θ (º)** | **As-prepared CeO_x_/C** | | **CeO_x_/C after S-absorption capacity test** | |
| --- | --- | --- | --- | --- | --- | --- |
|  |  |  | **B (º)** | **D_p_ (nm)** | **B (º)** | **D_p_ (nm)** |
| **CeO_2_** Cubic Fm-3m #ICDD 01-081-0792 | 1,1,1 | 28.54 | 2.02 | 4.1 | 1.51 | 5.4 |
|  | 2,0,0 | 33.05 | 2.52 | 3.4 | 1.51 | 5.5 |
|  | 2,2,0 | 47.48 | N.D. |  | 1.51 | 5.7 |
|  | 3,1,1 | 56.33 | 2.02 | 4.5 | 1.76 | 5.1 |
|  | 4,0,0 | 69.40 | 2.02 | 4.8 | 2.02 | 4.8 |
|  | 3,3,1 | 76.69 | 2.52 | 4.0 | 1.51 | 6.7 |
|  | 4,2,0 | 79.06 | 2.52 | 4.1 | 1.51 | 6.8 |
|  | **Average** | |  | **4.1** |  | **5.7** |
| **Ce_2_S_3_** Cubic I-43d #ICDD 00-027-0104 | 3,1,0 | 32.77 |  |  | 1.51 | 5.5 |
|  | 4,0,0 | 41.81 |  |  | 1.01 | 8.4 |
|  | **Average** | |  |  |  | **6.1** |


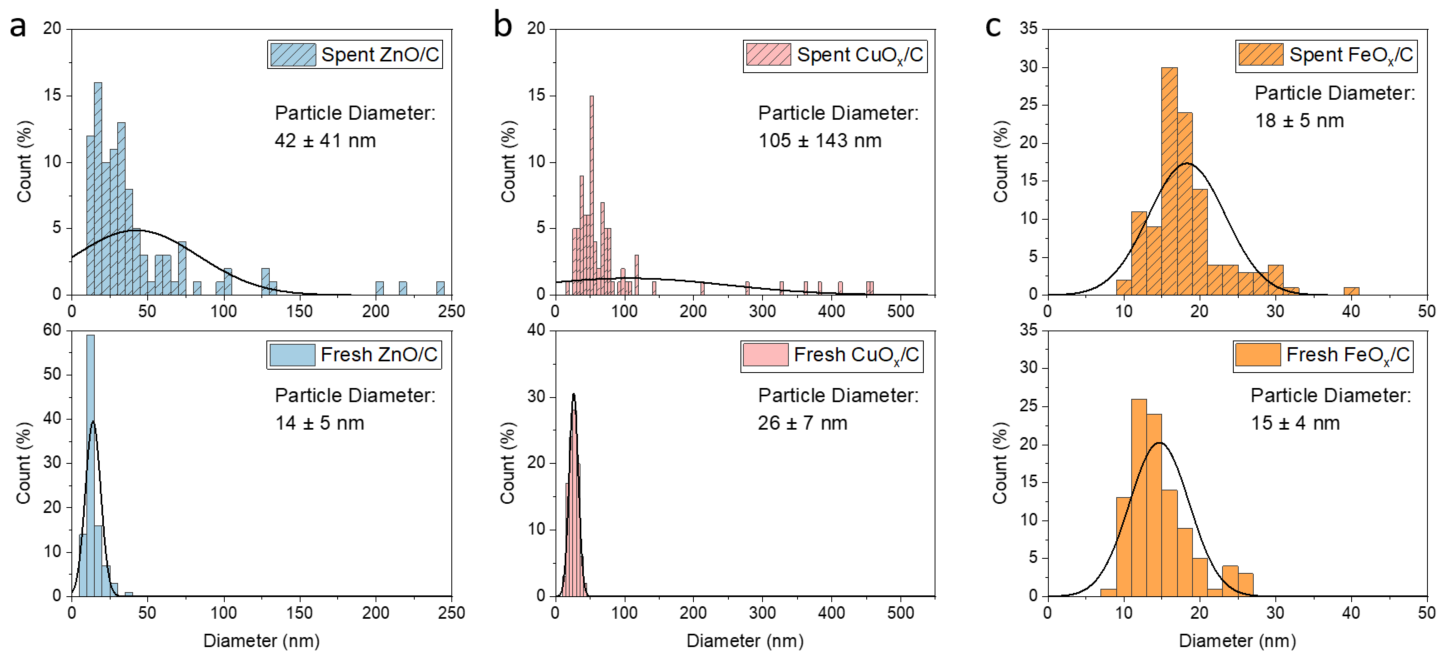


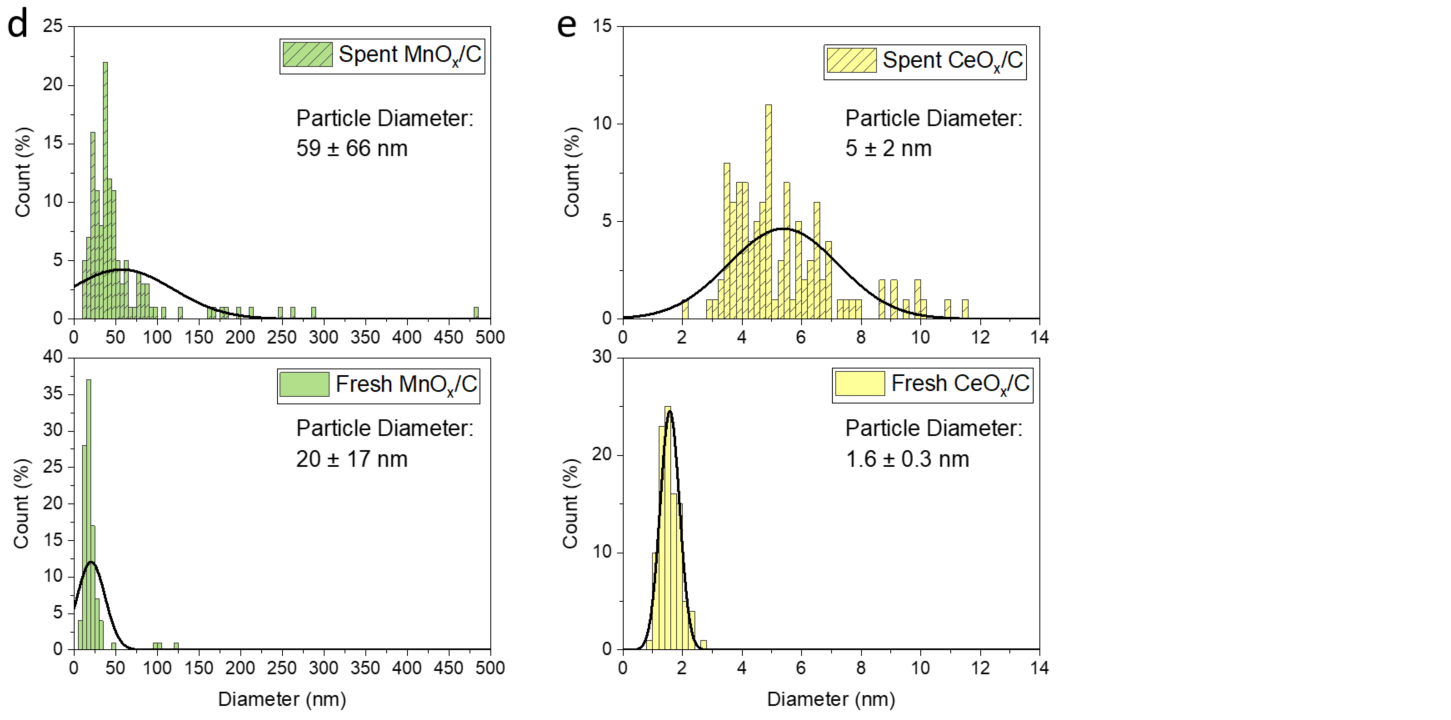


***Figure S5 – Particle size distribution (PSD) histograms of fresh and spent a) ZnO/C, b) CuO_x_/C, c) FeO_x_/C, d) MnO_x_/C, e) CeO_x_/C obtained from TEM images. The spent materials correspond to materials after HTG S-absorption capacity tests.***

a


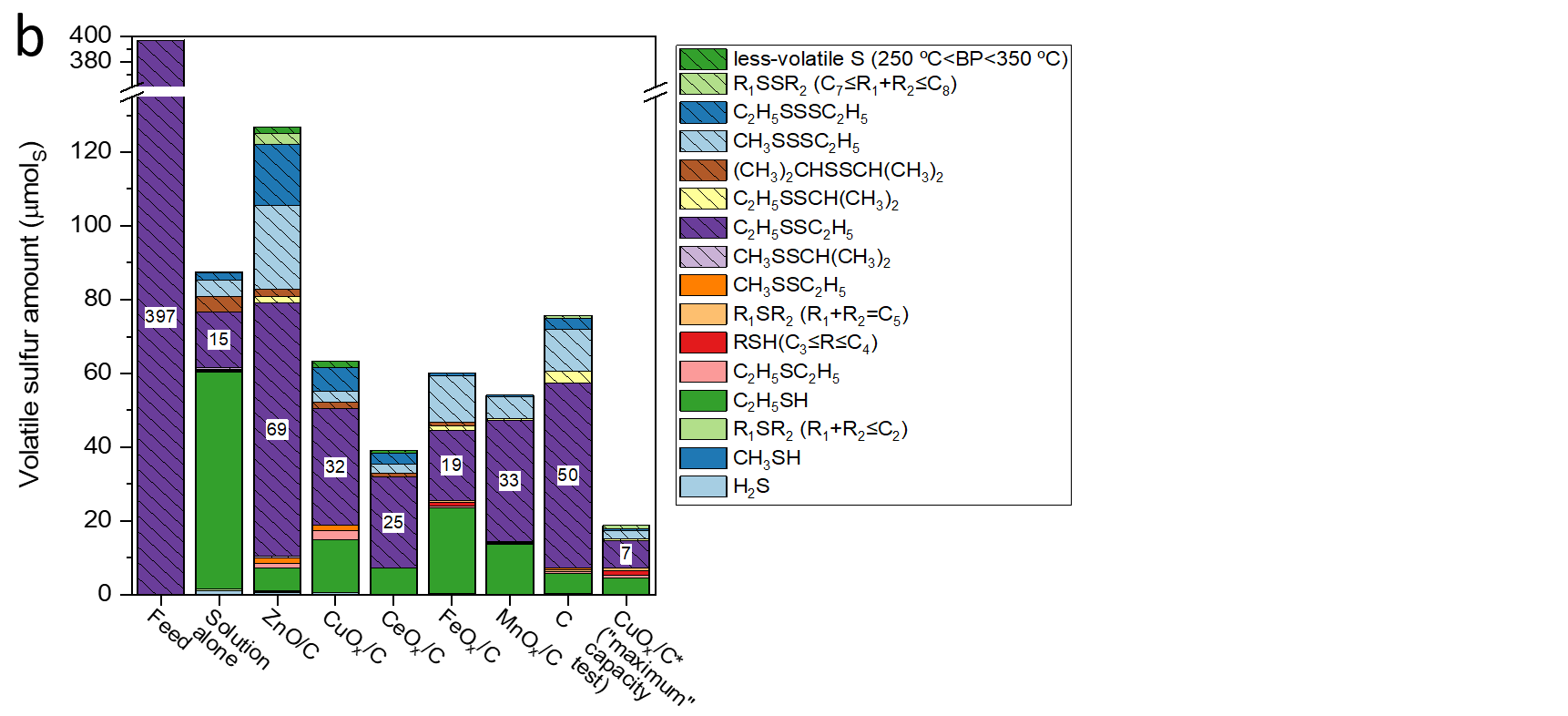


Figure S6 – a) The composition of gas product and b) distribution of volatile organosulfur compounds (bp ≤ 350 ºC) in the liquid product after HTG “S-absorption capacity” tests without (solution alone) and with MO_x_/Cs and C S-absorbents. *- “maximum” S-absorption capacity test using the same DEDS feed but with different conditions of M:S = 1 mol_M_ mol_S_^-1^ with HTG reaction time 15 h.


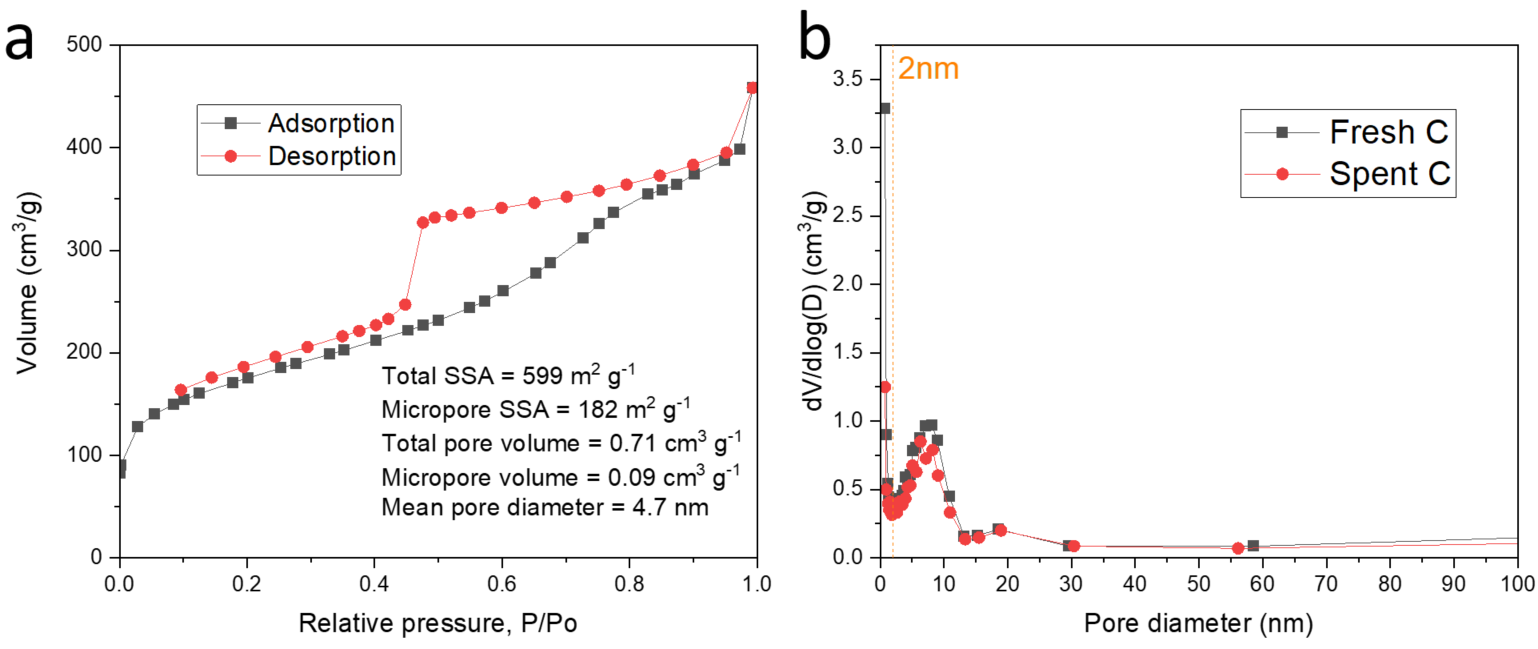


Figure S7 – a) N_2_ adsorption–desorption isothermal curves of the spent C after the S-absorption capacity test. b) Comparison of the pore size distribution of the fresh C and the spent C after the S-absorption capacity test.


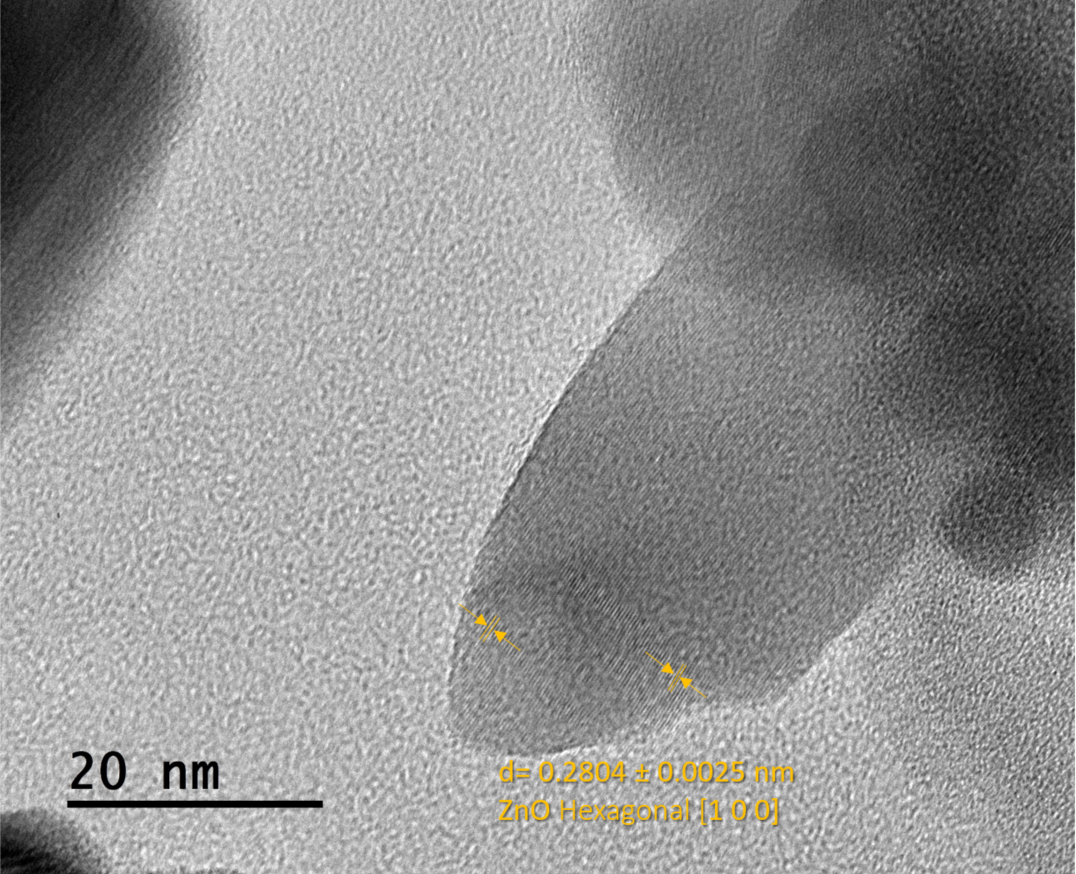


Figure S8 – HRTEM image focusing on a rod-like particle of spent ZnO/C after the S-absorption capacity test


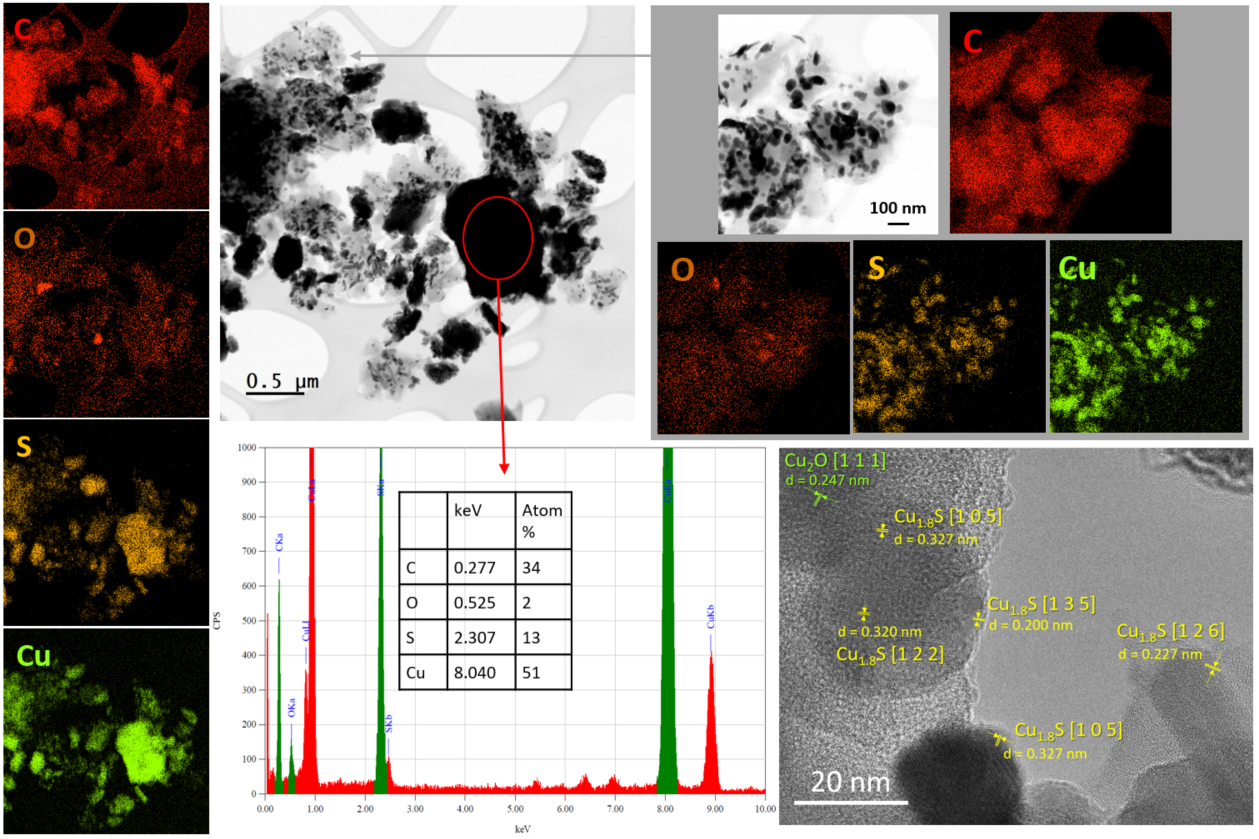


Figure S9 - TEM images and STEM-EDX elemental mapping of spent CuO_x_/C after “maximum” S-absorption capacity test with DEDS model feed.


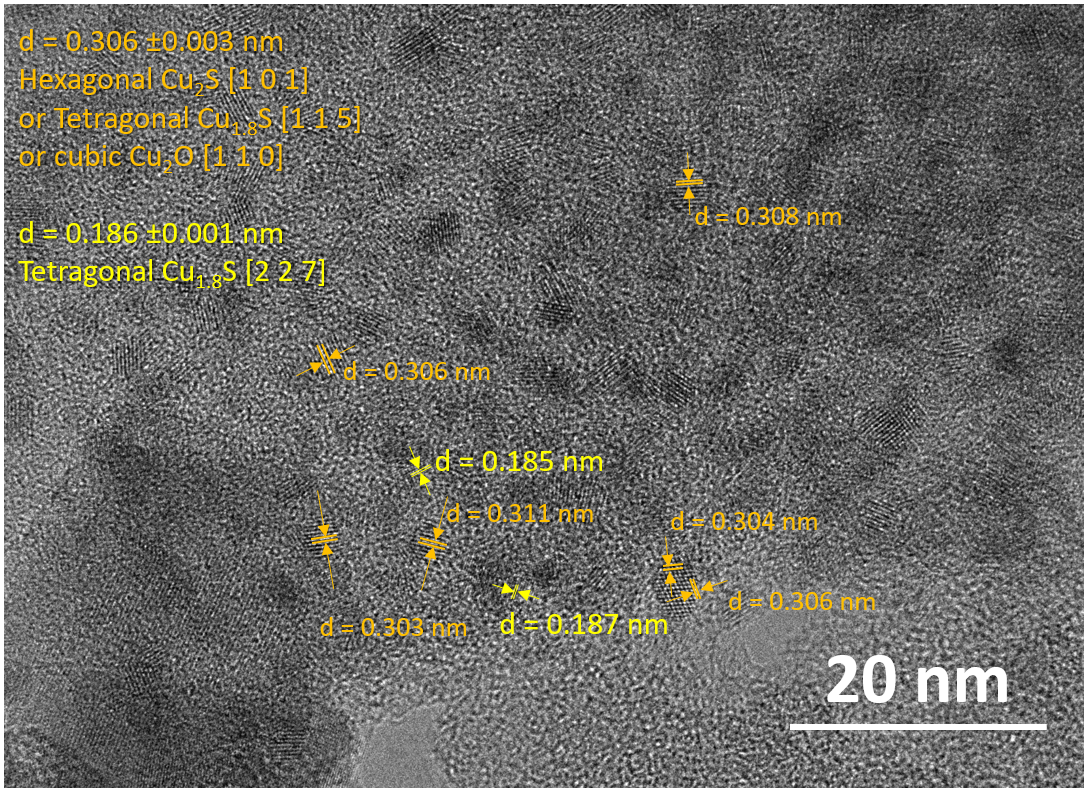


Figure S10 – HR-TEM image focusing on nanoparticle distributed area of spent CuO_x_/C after the “maximum” S-absorption test


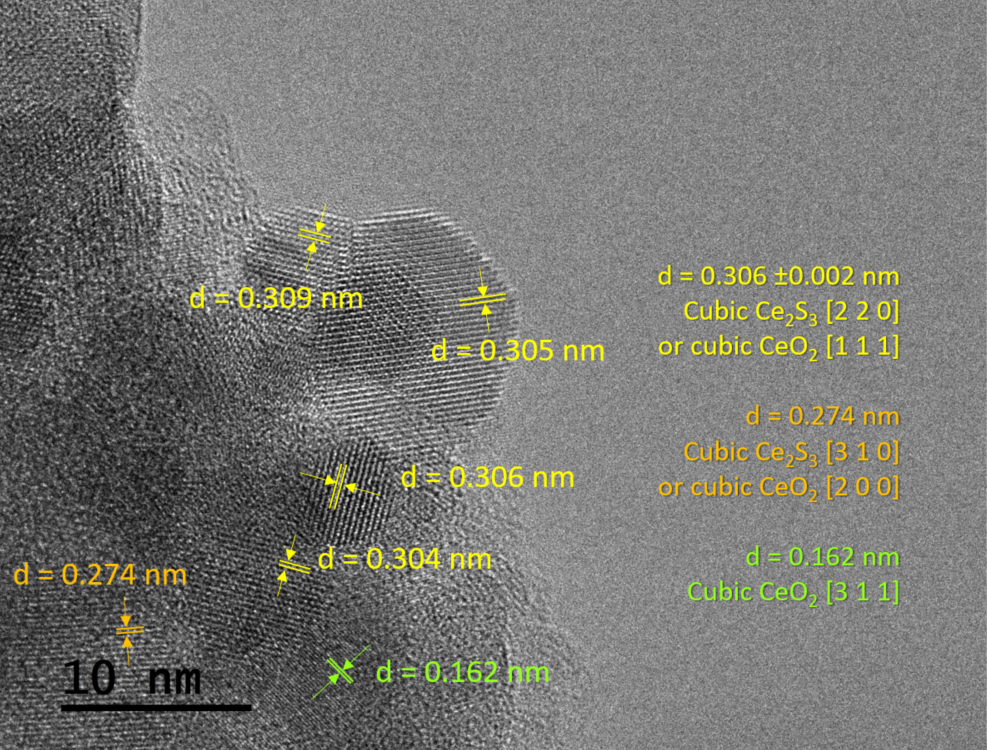


Figure S11 – HR-TEM image focusing on nanoparticle distributed area of spent CeO_x_/C after S-absorption capacity test

# Appendix: Details in Calculations of MO_x_ solubility in water

The concentrations of dissolved MO_x_ (ZnO, Cu_2_O, Cu, Fe_3_O_4_, MnO, and CeO_2_) in pure neutral water from liquid to supercritical state were calculated from the equilibrium constants (*K_eq_*) of the dissolution reactions listed in Table S2, following the revised Helgeson−Kirkham−Flowers (R-HKF) thermodynamic model applied by Shock et al.[1] and Jocz et al.[2]. Within the temperature and pressure (T&P) regime of a representative HTG reaction in the batch reactor carried out in this experimental work (see Figure S12), 8 different T&P conditions with corresponding water densities were extracted as listed in Table S4, which were used as different set of conditions for MO_x_ solubility calculation.

Figure S12 – A representative T&P profile when carrying out HTG reactions in the batch reactor with 15 min reaction time (start counting when T reached 450 °C) and the corresponding water density (ρ_water_) and water state obtained from NIST [3]. The phases indicated are the dominating one in the highlighted areas.

The equilibrium constant (*K_eq_*) and the change in free energy of the dissolution reactions (*ΔG_rxn_*) were calculated by Equation S1 and 2, where *R* is the universal gas constant, $v_{j}$is the stoichiometric coefficient of species *j* in the reaction (positive for products and negative for reactants) and ${\Delta G}_{f}\left( T,\rho_{H_{2}O} \right)_{j}$is the apparent standard partial molar Gibbs free energy of the formation of species *j* at specific temperature *T* and water density $\rho_{H_{2}O}$.

Table S2 - Dissolution reactions used to calculate MO_x_ solubility in pure neutral water at different states.

| Reactant/product(s) | Reaction equation |
| --- | --- |
| ZnO/Zn^2+^ | ZnO + H_2_O → Zn^2+^ + 2OH^-^ |
| ZnO/Zn(OH)^+^ | ZnO + H_2_O → Zn(OH)^+^ + OH^-^ |
| ZnO/HZnO_2_^-^ | ZnO + H_2_O → HZnO_2_^-^ + H^+^ |
| ZnO/ZnO_2_^2-^ | ZnO + H_2_O → ZnO_2_^2-^ + 2H^+^ |
| Cu_2_O/Cu^+^ | Cu_2_O + H_2_O → 2Cu^+^ + 2OH^-^ |
| Cu/Cu^+^ | Cu + H_2_O → Cu^+^ + OH^-^ + ½ H_2_ (aq) |
| Fe_3_O_4_/Fe^2+^, Fe^3+^ | Fe_3_O_4_ + 4H_2_O → Fe^2+^ + 2Fe^3+^ + 8OH^-^ |
| Fe_3_O_4_/FeOH^+^, FeOH^2+^ | Fe_3_O_4_ + 4H_2_O → FeOH^+^ + 2FeOH^2+^ + 5OH^-^ |
| Fe_3_O_4_/HFeO_2_^-^, FeO_2_^-^ | Fe_3_O_4_ + 2H_2_O → HFeO_2_^-^ + 2FeO_2_^-^ + 3H^+^ |
| MnO/Mn^2+^ | MnO + H_2_O → Mn^2+^ + 2OH^-^ |
| MnO/Mn(OH)^+^ | MnO + H_2_O → Mn(OH)^+^ + OH^-^ |
| MnO/HMnO_2_^-^ | MnO + H_2_O → HMnO_2_^-^ + H^+^ |
| MnO/MnO_2_^2-^ | MnO + H_2_O → MnO_2_^2-^ + 2H^+^ |
| CeO_2_/Ce^4+^ | CeO_2_ + 2H_2_O → Ce^4+^ + 4OH^-^ |
| CeO_2_/CeOH^3+^ | CeO_2_ + 2H_2_O → CeOH^3+^ + 3OH^-^ |
| CeO_2_/Ce(OH)_2_^2+^ | CeO_2_ + 2H_2_O → Ce(OH)_2_^2+^ + 2OH^-^ |

${\Delta G}_{f}\left( T,\rho_{H_{2}O} \right)_{j}$ of solid species was obtained from Equation S3. *ΔG_j_^0^* and *S_j_^0^* are respectively the standard partial molar Gibbs free energy of formation and molar entropy of species *j* at the standard reference temperature *T_0_* (298.15 K) and pressure *P_0_* (1 bar), which can be obtained from the thermochemical databases [4] and [5]. *C_p_(T)* is the molar heat capacity and can be described by the polynomial function of *T* given in Equation S4, where the coefficients *a – e* were fitted from the tabulated *C_p_(T)* data [4] [5]. Table S3 summarizes those thermodynamic parameters of MO_x_ solid species.

| $K_{eq}{(T,\rho_{H_{2}O})}_{i}=e^{\frac{-\Delta G_{rxn}{(T,\rho_{H_{2}O})}_{i}}{RT}}$ | Equation S1 |
| --- | --- |
| ${\Delta G}_{rxn}\left( T,\rho_{H_{2}O} \right)=\sum_{j} v_{j}{\Delta G}_{f}\left( T,\rho_{H_{2}O} \right)_{j}$ | Equation S2 |
| $\Delta G_{f}\left( T,\rho_{H_{2}O} \right)_{j}=\Delta G_{j}^{0}-\int_{T_{0}}^{T_{1}} S\left( T \right)dT+\int_{P_{0}}^{P_{1}} VdP=\Delta G_{j}^{0}+V(P_{1}-P_{0})-\int_{T_{0}}^{T_{1}} {[S_{j}}^{0}+\int_{T_{0}}^{T_{1}} \frac{C_{P}(T)}{T}dT]dT$ | Equation S3 |
| $C_{p}\left( T \right)=a+bT+{cT}^{2}+{dT}^{3}+{eT}^{-2}$ | Equation S4 |

$\boldsymbol{\Delta G}_{\boldsymbol{f}}\left( \boldsymbol{T,}\boldsymbol{\rho}_{\boldsymbol{H}_{\boldsymbol{2}}\boldsymbol{O}} \right)_{\boldsymbol{j}}$ of water at different states was also calculated by Equation S3 with the results listed in Table S4. ΔG_H2O_^0^ and S_H2O_^0^ of water at the standard state is respectively -237.2 kJ mol^-1^ and 69.95 J mol^-1^ K^-1^ from NIST[3].

$\boldsymbol{\Delta G}_{\boldsymbol{f}}\left( \boldsymbol{T,}\boldsymbol{\rho}_{\boldsymbol{H}_{\boldsymbol{2}}\boldsymbol{O}} \right)_{\boldsymbol{j}}$ of ions and dissolved H_2_ and O_2_ was calculated using R-HKF equation [1] (Equation S5), where Ψ and Θ are solvent parameters equal to 2600 bar and 228 K, respectively. a_1_, a_2_, a_3_, a_4_, c_1_, and c_2_ represent species-dependent non-solvation parameters that can be obtained from [6]. $\boldsymbol{\epsilon}_{\left( \boldsymbol{P}\boldsymbol{,}\boldsymbol{T} \right)}$ is the dielectric constant of water and can be described as a function of T at specific pressure given in Equation S6, where the coefficients b_1_ – b_4_ were fitted from the tabulated $\boldsymbol{\epsilon}_{\left( \boldsymbol{P}\boldsymbol{,}\boldsymbol{T} \right)}$ data [7]. $\boldsymbol{\omega}_{\boldsymbol{j}\left( \boldsymbol{P}\boldsymbol{,}\boldsymbol{T} \right)}$ is the conventional Born coefficient. For ion j, it was calculated by Equation S7, where η is the HKF model constant equals to 694657Å J mol^-1^, Z_j_ is the charge, $\boldsymbol{\omega}_{\boldsymbol{H}^{\boldsymbol{+}}}^{\boldsymbol{ads}}$is the absolute Born coefficient of the reference ion H^+^ at 298.15 K and 1 bar with the value 225.39208 kJ mol^-1^. r_e,j_ is the effective electrostatic radius varied by T and P while r_x,j_ is the crystal radius of ion j and can be obtained from thermodynamic database or calculated by Equation S8 using the effective electrostatic radius r^0^_e,j_ at T_0_ (298.15 K) and P_0_ (1 bar) [1]. k_z_ represents a charge-dependent constant equal to 0 Å for anions and 0.94 Å for cations. Calculation of r^0^_e,j_ using the standard molar entropy S_j_^0^ is given as Equation S9. $\boldsymbol{g}_{\boldsymbol{(P,T)}}$denotes a solvent (water) function of temperature and pressure which can be calculated by the Equation (32) and (33) from [8]. $\boldsymbol{Y}_{\left( \boldsymbol{P}\boldsymbol{, T} \right)}$ is the Born function of water calculated by Equation S10.

Since the effective charge and effective electrostatic radius of neutral aqueous species, such as dissolved H_2_ and O_2_, cannot be assessed properly, their $\boldsymbol{\omega}_{\boldsymbol{j}}$ can be regarded in a first approximation as T/P-independent parameters[9]. It follows from the revised HKF model that the solvation contributions to the standard partial molar volumes ($\boldsymbol{\Delta}\boldsymbol{V}_{\boldsymbol{S}}^{\boldsymbol{0}}$) as described in Equation S11. The T&P dependence of $\boldsymbol{\Delta}\boldsymbol{V}_{\boldsymbol{n}}^{\boldsymbol{0}}$ can be expressed as Equation S12. $\boldsymbol{Q}_{\left( \boldsymbol{P}\boldsymbol{, T} \right)}$ is also a Born function similar to $\boldsymbol{Y}_{\left( \boldsymbol{P}\boldsymbol{, T} \right)}$ and is expressed as Equation S13. Those solvent parameters at different T and P are calculated and listed in Table S4. Table S5 lists those standard thermodynamic and HKF parameters of aqueous inorganic ions and dissolved H_2_ and O_2_ included in this study.

${\Delta G}_{f}\left( T,\rho_{H_{2}O} \right)_{j}$ of H^+^ was set as the reference for all aqueous species and equal to 0 at all conditions while ${\Delta G}_{f}\left( T,\rho_{H_{2}O} \right)_{j}$ of OH^-^ was calculated using Equation S14.

| $\Delta G_{f}\left( T,\rho_{H2O} \right)_{j}={\Delta G}_{f}^{0}-S^{0}\left( T_{1}-T_{0} \right)-c_{1}\left[ T_{1}ln\left( \frac{T_{1}}{T_{0}} \right)-T_{1}+T_{0} \right]+a_{1}\left( P_{1}-P_{0} \right)+a_{2}\ln\left( \frac{\psi+P_{1}}{\psi+P_{0}} \right)-c_{2}\left\{ \left[ \left( \frac{1}{T_{1}-\Theta} \right)-\left( \frac{1}{T_{0}-\Theta} \right) \right]\left( \frac{\Theta-T_{1}}{\Theta} \right)-\frac{T_{1}}{\Theta^{2}}\ln\left[ \frac{T_{0}\left( T_{1}-\Theta\right)}{T_{1}\left( T_{0}-\Theta\right)} \right] \right\}+\left( \frac{1}{T_{1}-\Theta} \right)\left[ a_{3}\left( P_{1}-P_{0} \right)+a_{4}\ln\left( \frac{\psi+P_{1}}{\psi+P_{0}} \right) \right]+\omega_{\left( P_{1},T_{1} \right)}\left( \frac{1}{\epsilon_{\left( P_{1},T_{1} \right)}}-1 \right)-\omega_{\left( P_{0},T_{0} \right)}\left( \frac{1}{\epsilon_{\left( P_{0},T_{0} \right)}}-1 \right)+\omega_{\left( P_{0},T_{0} \right)}Y_{\left( P_{0},T_{0} \right)}\left( T_{1}-T_{0} \right)$ | Equation S5 |
| --- | --- |
| $\epsilon\left( T \right)=b_{1}+b_{2}T+{b_{3}T}^{2}+{b_{4}T}^{3}$ | Equation S6 |
| $\omega_{j\left( P,T \right)}=\frac{\eta Z_{j}^{2}}{r_{e,j}}-Z_{j}\omega_{H^{+}}^{ads}=\frac{\eta Z_{j}^{2}}{r_{x,j}+\left\vert Z_{j} \right\vert(k_{z}+g_{(P,T)})}-Z_{j}\omega_{H^{+}}^{ads}$ | Equation S7 |
| $r_{x,j}=r_{e,j}^{0}-\left\vert Z_{j} \right\vert\left[ k_{z}+g_{\left( P_{0},T_{0} \right)} \right]$ | Equation S8 |
| $r_{e,j}^{0}=\frac{Z_{j}^{2}(\eta Y_{(P_{0},T_{0})}-100)}{S_{j}^{0}-71.5\left\vert Z_{j} \right\vert}$ | Equation S9 |
| $Y_{\left( P,T \right)}=\frac{1}{\epsilon}{(\frac{\partial ln\epsilon}{\partial T})}_{P}=\frac{1}{\epsilon}\times{\frac{dln(b_{1}+b_{2}T+b_{3}T^{2}+b_{4}T^{3})}{dT}=\frac{1}{\epsilon}\times\frac{b_{2}+{2b}_{3}T+{3b}_{4}T^{2}}{b_{1}+b_{2}T+b_{3}T^{2}+b_{4}T^{3}}}$ | Equation S10 |
| $\Delta V_{S}^{0}=V^{0}-{\Delta V}_{n}^{0}=-\omega_{j}Q$ | Equation S11 |
| ${\Delta V}_{n}^{0}=a_{1}+a_{2}\left( \frac{1}{\psi+P} \right)+\left[ a_{3}+a_{4}\left( \frac{1}{\psi+P} \right) \right](\frac{1}{T-\Theta})$ | Equation S12 |
| $Q_{\left( P,T \right)}=\frac{1}{\epsilon}{(\frac{\partial ln\epsilon}{\partial P})}_{T}=\frac{1}{\epsilon}\times{\frac{dln(c_{1}+c_{2}P+c_{3}P^{2}+c_{4}P^{3})}{dP}=\frac{1}{\epsilon}\times\frac{c_{2}+{2c}_{3}P+{3c}_{4}P^{2}}{c_{1}+c_{2}P+c_{3}P^{2}+c_{4}P^{3}}}$ | Equation S13 |
| $\Delta G_{f,{OH}^{-}}=-RTln\left( K_{W} \right)+{\Delta G}_{f,H_{2}O}-\Delta G_{f,H^{+}}$ | Equation S14 |

Thus, based on the above Equation S2 – 13, *ΔG_rxn_* and *K_eq_* of the targeted dissolution reactions of MO_x_ under the 8 different states of water were calculated as listed in Table S6.

To further calculate the molar concentration *m_j_* (mol kg^-1^, i.e. per kg of water) of all the dissolved species in water at specific condition, Equation S15 relating the equilibrium constants to the thermodynamic activity was used, where *m_Θ_* is the reference concentration equals to 1 mol kg^-1^, *a_j_* and *γ_j_* is respectively the thermodynamic activity and activity coefficient which is taken to be unity for non-charged species including neutral molecules, solid, and H_2_O. *γ_j_* of charged aqueous species was calculated using the Davies extension of the Debye-Hückel equation [10] as shown in Equation S16, where *Z_j_* is the charge on the *j*^th^ species, *A_Φ_* is the Debye−Hückel parameter calculated from Equation S17, and *I* is the ionic strength of the solution calculated from Equation S18. Extensions of the Debye−Hückel equation work for aqueous solution with low ionic strength (< 0.2 mol kg^-1^) which matches the hydrothermal systems. For a solution of N total aqueous species (H^+^, OH^−^, and N–2 dissolved species from the MO_x_) with a concentration-dependent ionic strength, there are N + 1 unknown variables (N concentrations m_j_ plus ionic strength I), and so there must be N + 1 equations to calculate these unknowns. Combining N-2 equilibrium expressions (Equation S15) for all dissolution reactions (listed in Table S2) of the target MO_x_, the equilibrium equation for the ion product of water, the definition of ionic strength in Equation S18, and lastly the expression of charge neutrality in Equation S19, the N+1 unknowns were solved using Matlab’s nonlinear least-squares solver function (lsqnonlin) with “levenberg-marquardt” algorithm. Because the species concentrations could be very low (< 10^-8^ mol kg^-1^), some below the error tolerance of the solver, logarithm was taken for both sides of the N+1 equations before solving.

| $K_{eq}{(T,\rho_{H_{2}O})}_{i}=\prod_{j} a_{j}^{v_{j}}=\prod_{j} (\frac{\gamma_{j}m_{j}}{m_{\Theta}})^{v_{j}}$ | Equation S15 |
| --- | --- |
| $ln\gamma_{j}=-\frac{Z_{j}^{2}A_{\Phi}I^{\frac{1}{2}}}{1+I^{\frac{1}{2}}}+0.2A_{\Phi}I$ | Equation S16 |
| $A_{\Phi}=\frac{1.8246\times{10}^{6}(\rho_{H_{2}O}/1.00)^{1/2}}{(\epsilon T)^{3/2}}$ | Equation S17 |
| $I=\frac{1}{2}\sum_{j}^{N} m_{j}{Z_{j}}^{2}$ | Equation S18 |
| $\sum_{j}^{N} m_{j}Z_{j}=0$ | Equation S19 |

The calculated solubility of ZnO, Cu_2_O, Cu, Fe_3_O_4_, MnO, and CeO_2_ in water are listed in Table S7.

Table S3 – Standard thermodynamic parameters of solid MO_x_ studied in this paper

|  | *ΔG_j_^0^* ^a^  (J mol^-1^) | *ΔH_j_^0^* ^a^  (J mol^-1^) | *S_j_^0^* ^a^  (J mol^-1^ K^-1^) | *V_j_^0^* ^a^  (cm^3^ mol^-1^) | *C_P_^0^* coefficients ^b^ | | | | |
| --- | --- | --- | --- | --- | --- | --- | --- | --- | --- |
|  |  |  |  |  | *a* | *b* | *c* | *d* | *e* |
| ZnO | -320476 | -350460 | 43.64 | 14.51 | 45.323 | 0.0074 | -8.4E-08 | 3.8E-11 | -570843 |
| Cu_2_O | -147880 | -170707 | 92.34 | 23.85 | 63.211 | 0.0237 | -7.7E-06 | 2.7E-09 | -632487 |
| Cu | 0 | 0 | 33.16 | 23.85 | 22.749 | 0.0100 | -8.6E-06 | 4.59E-09 | -56246.6 |
| Fe_3_O_4_ | -1015227 | -1118383 | 146.15 | 44.78 | -351.625 | 2.3639 | -0.00373 | 2.16E-06 | 6410730 |
| MnO | -362898 | -385221 | 59.71 | 13.21 | 46.925 | 0.0078 | -4.6E-09 | 4.05E-12 | -456967 |
| CeO_2_ | -1025379 | -1088677 | 62.30 | 23.86 | 70.142 | 0.0092 | 5.41E-08 | -2.2E-11 | -1001226 |
| ^a^ – obtained from [4]. ^b^ – *C_P_^0^* coefficients *a - e* were obtained by fitting *C_P_(T)* (J mol^-1^ K^-1^) data with the range 298.15 – 1000 K from [4]. | | | | | | | | | |

Table S4 – Thermodynamic and solvent-related parameters of water at selected 8 different conditions

| water state | *T*  (°C) | *P*  (bar) | *ρ*  (g ml^-1^) | *∆G_f_* ^a^  (J mol^-1^) | *є* ^b^ | dielectric constant parameters ^c^ | | | | *g* ^d^  (Å) | *Y* ^e^  (K^-1^) | *logK_W_* ^f^  [log(mol kg^-1^)^2^] | *A_Ф_* ^g^ |
| --- | --- | --- | --- | --- | --- | --- | --- | --- | --- | --- | --- | --- | --- |
|  |  |  |  |  |  | *b_1_* | *b_2_* | *b_3_* | *b_4_* |  |  |  |  |
| liquid | 50 | 35 | 0.990 | -242052 | 69.44 | 279.49 | -1.070 | 1.60E-03 | -9.14E-07 | 0 | -6.61E-05 | -13.263 | 0.540 |
| liquid | 200 | 51 | 0.867 | -273001 | 35.43 | 280.75 | -1.080 | 1.63E-03 | -9.38E-07 | -1.293E-04 | -1.37E-04 | -11.268 | 0.783 |
| liquid | 300 | 94 | 0.714 | -294752 | 20.02 | 289.81 | -1.146 | 1.79E-03 | -1.06E-06 | -3.475E-03 | -3.54E-04 | -11.392 | 1.254 |
| liquid | 350 | 167 | 0.576 | -305727 | 13.27 | 287.12 | -1.123 | 1.72E-03 | -1.01E-06 | -2.108E-02 | -7.99E-04 | -12.278 | 1.842 |
| vapor | 375 | 217 | 0.182 | -309817 | 2.20 | 282.76 | -1.101 | 1.70E-03 | -1.02E-06 | -3.577E-01 | -9.43E-03 | -19.101 | 14.471 |
| supercritical | 400 | 244 | 0.155 | -314882 | 2.50 | 297.07 | -1.192 | 1.89E-03 | -1.12E-06 | -4.146E-01 | -1.29E-02 | -19.857 | 10.415 |
| supercritical | 425 | 267 | 0.144 | -320191 | 2.30 | 294.71 | -1.173 | 1.83E-03 | -1.08E-06 | -4.018E-01 | -4.39E-02 | -20.090 | 10.756 |
| supercritical | 450 | 300 | 0.143 | -325589 | 2.16 | 273.45 | -1.022 | 1.49E-03 | -8.31E-07 | -4.205E-01 | -2.07E-01 | -19.958 | 11.195 |
| ^a^ – calculated from Equation S3 in this paper. ^b^ – obtained or fitted from [7]. ^c^ – fitted from [7]. ^d^ – solvent function, obtained or calculated by the Equation (32) and (33) from [8]. ^e^ – Born function, calculated by Equation S10 in this paper. ^f^ – logarithm ion product of water (base 10), calculated by the Equation (4) from [11]. ^g^ – Debye-Hückel parameter, calculated by Equation S17 in this paper. | | | | | | | | | | | | | |

Table S5 – Standard thermodynamic and HKF parameters of aqueous inorganic ions used to model MO_x_ dissolution in water.

| ions | *ΔG_j_^0^* ^a^  (J mol^-1^) | *ΔH_j_^0^* ^a^  (J mol^-1^) | *S_j_^0^* ^a^  (J mol^-1^ K^-1^) | *V_j_^0^* ^a^  (cm^3^ mol^-1^) | *C_P_^0^* ^a^  (J mol^-1^ K^-1^) | r_x_ ^b^  (Å) | species-dependent non-solvation parameters ^a^ | | | | | | *ω_(P0,T0)_* ^a^  (J mol^-1^) |
| --- | --- | --- | --- | --- | --- | --- | --- | --- | --- | --- | --- | --- | --- |
|  |  |  |  |  |  |  | *a_1_ ^c^* | *a_2_ ^d^* | *a_3_ ^e^* | *a_4_ ^f^* | *c_1_ ^g^* | *c_2_ ^h^* |  |
| Zn^2+^ | -147277 | -153385 | -109.62 | -24.30 | -22.30 | 0.71 | -0.4467 | -3735.89 | 25.640 | -100826 | 78.408 | -224681 | 609776 |
| Zn(OH)^+^ | -339699 | -363966 | 62.76 | -5.30 | 41.84 | 1.00 | 0.4811 | -2078.49 | 32.173 | -107675 | 62.888 | -41735 | 136398 |
| HZnO_2_^-^ | -463252 | -595676 | -66.94 | -13.40 | 83.68 | 1.25 | 0.2353 | -2679.73 | 34.555 | -105190 | 146.806 | 43488 | 781111 |
| ZnO_2_^2-^ | -390325 | -552706 | -167.36 | -26.40 | -62.76 | 2.40 | -0.2326 | -3821.96 | 39.037 | -100470 | 136.330 | -254806 | 1598957 |
| Cu^+^ | 49998.8 | 71680.29 | 40.58 | -8.00 | 57.32 | 0.68 | 0.3376 | -2428.39 | 33.541 | -106232 | 74.991 | -10201 | 169285 |
| Fe^2+^ | -91504.1 | -92257.2 | -105.86 | -22.20 | -33.05 | 0.73 | -0.3292 | -4057.18 | 39.948 | -99495.5 | 61.865 | -194292 | 601743 |
| Fe^3+^ | -17238.1 | -49580.4 | -277.40 | -37.00 | -77.82 | 0.70 | -1.0149 | -5730.45 | 46.501 | -92579.4 | 79.688 | -285487 | 1079974 |
| FeOH^+^ | -275516 | -326687 | -41.84 | -16.50 | 62.76 | 0.41 | -0.1072 | -3516.19 | 37.847 | -101734 | 89.577 | 874 | 293006 |
| FeOH^2+^ | -241835 | -292880 | -106.27 | -24.90 | -34.31 | 0.73 | -0.4838 | -4435.42 | 41.454 | 611290.8 | -19.685 | -196849 | 601743 |
| HFeO_2_^-^ | -399154 | -525929 | -62.76 | -12.90 | 92.05 | 1.27 | 0.2624 | -2612.49 | 34.269 | -105470 | 151.303 | 60534 | 776718 |
| FeO_2_^-^ | -368192 | -463169 | 44.35 | 0.90 | -235.56 | 1.80 | 0.9973 | -820.15 | 27.272 | -112880 | -55.734 | -606797 | 613458 |
| Mn^2+^ | -230538 | -221334 | -67.78 | -17.10 | -17.15 | 0.88 | -0.0425 | -3348.87 | 36.987 | -102424 | 69.736 | -161908 | 586011 |
| Mn(OH)^+^ | -407103 | -446851 | 1.26 | -11.90 | 36.40 | 0.60 | 0.1344 | -2924.66 | 35.489 | -104177 | 68.197 | -52815 | 228614 |
| HMnO_2_^-^ | -506264 | -627182 | -38.07 | -9.70 | -13.81 | 1.36 | 0.4332 | -2195.76 | 32.636 | -107190 | 85.879 | -155088 | 739940 |
| MnO_2_^2-^ | -429278 | -557727 | -63.60 | -13.00 | -299.16 | 2.77 | 0.4807 | -2079.99 | 32.190 | -107671 | -16.893 | -736346 | 1439631 |
| Ce^4+^ | -507519 | -576137 | -418.80 | -53.30 | 0.42 | 0.79 | -1.7904 | -7625.21 | 53.972 | -84747 | 168.530 | -126110 | 1546574 |
| CeOH^3+^ | -749350 | -835147 | -243.20 | -4.40 | 121.63 | 0.80 | 0.8837 | -1098.08 | 28.374 | -111734 | 193.000 | 120794 | 1051074 |
| Ce(OH)_2_^2+^ | -1012500 | -1137903 | -142.90 | -0.56 | -91.54 | 0.60 | 0.9600 | -911.70 | 27.642 | -112504 | 33.420 | -313431 | 671493 |
| H_2_ (aq)^i^ | 17723 | -4184 | 57.7 | 25.2 | 166.94 | -- | 2.1517 | 1998.20 | 16.204 | -124533 | 115.58 | 213091 | -87446 |
| O_2_ (aq)^i^ | 16544 | -12134 | 109.0 | 30.38 | 234.30 | -- | 2.4221 | 2658.35 | 13.610 | -127265 | 147.92 | 350310 | -164975 |
| ^a^ – obtained from the Table 1, 4 and 5 from [6]. ^b^ – calculated from Equation S8 in this paper.  ^c^ – unit J mol^-1^ bar^-1^.  ^d^ – unit J mol^-1^.  ^e^ – unit J K mol^-1^ bar^-1^.  ^f^ – unit J K mol^-1^.  ^g^ – unit J mol^-1^ K^-1^.  ^h^ – unit J K mol^-1^*.* ^i^ – aqueous (aq) | | | | | | | | | | | | | |

Table S6 – Calculated ΔG_rxn_ and log_10_(K_eq_) of the targeted dissolution reactions of MO_x_ in pure neutral water at 8 different states.

| Dissolution reaction ^a^ | 50 °C, 35 bar | | 200 °C, 51 bar | | 300 °C, 94 bar | | 350 °C, 167 bar | | 375 °C, 217 bar | | 400 °C, 244 bar | | 425 °C, 267 bar | | 450 °C, 300 bar | |
| --- | --- | --- | --- | --- | --- | --- | --- | --- | --- | --- | --- | --- | --- | --- | --- | --- |
|  | *ΔG_rxn_*^b^ | *log_10_ (K_eq_)* | *ΔG_rxn_* | *log_10_ (K_eq_)* | *ΔG_rxn_* | *log_10_ (K_eq_)* | *ΔG_rxn_* | *log_10_ (K_eq_)* | *ΔG_rxn_* | *log_10_ (K_eq_)* | *ΔG_rxn_* | *log_10_ (K_eq_)* | *ΔG_rxn_* | *log_10_ (K_eq_)* | *ΔG_rxn_* | *log_10_ (K_eq_)* |
| ZnO/Zn^2+^ | 99.1 | -16.0 | 133.5 | -14.7 | 179.3 | -16.3 | 220.6 | -18.5 | 458.3 | -36.9 | 389.7 | -30.2 | 459.4 | -34.4 | 486.2 | -35.1 |
| ZnO/Zn(OH)^+^ | 62.3 | -10.1 | 79.4 | -8.8 | 101.2 | -9.2 | 122.2 | -10.2 | 236.1 | -19.0 | 237.4 | -18.4 | 257.0 | -19.2 | 267.4 | -19.3 |
| ZnO/HZnO_2_^-^ | 102.0 | -16.5 | 148.8 | -16.4 | 186.4 | -17.0 | 208.2 | -17.5 | 380.8 | -30.7 | 294.4 | -22.8 | 344.1 | -25.7 | 369.4 | -26.7 |
| ZnO/ZnO_2_^2-^ | 177.6 | -28.7 | 245.1 | -27.1 | 305.5 | -27.8 | 341.9 | -28.7 | 678.6 | -54.7 | 488.5 | -37.9 | 591.0 | -44.2 | 639.0 | -46.2 |
| Cu_2_O/Cu^+^ | 170.1 | -27.5 | 178.9 | -19.7 | 203.7 | -18.6 | 235.8 | -19.8 | 467.1 | -37.6 | 454.2 | -35.2 | 493.2 | -36.9 | 510.2 | -36.9 |
| Cu/Cu^+^ | 52.5 | -8.5 | 67.8 | -7.5 | 115.1 | -10.5 | 139.2 | -11.7 | 254.8 | -20.5 | 248.6 | -19.3 | 268.3 | -20.1 | 277.1 | -20.0 |
| Fe_3_O_4_/Fe^2+^, Fe^3+^ | 597.8 | -96.6 | 769.2 | -84.9 | 979.8 | -89.3 | 1156.2 | -96.9 | 2070.0 | -166.8 | 1707.4 | -132.5 | 2031.8 | -152.0 | 2148.6 | -155.2 |
| Fe_3_O_4_/FeOH^+^, FeOH^2+^ | 434.6 | -70.2 | 577.3 | -63.7 | 736.9 | -67.2 | 864.7 | -72.5 | 1471.6 | -118.6 | 1319.9 | -102.4 | 1503.7 | -112.5 | 1583.6 | -114.4 |
| Fe_3_O_4_/HFeO_2_^-^, FeO_2_^-^ | 367.3 | -59.4 | 466.6 | -51.5 | 561.3 | -51.2 | 622.9 | -52.2 | 1142.4 | -92.1 | 956.5 | -74.2 | 1074.0 | -80.4 | 1144.4 | -82.7 |
| MnO/Mn^2+^ | 57.6 | -9.3 | 88.1 | -9.7 | 131.1 | -11.9 | 171.6 | -14.4 | 412.9 | -33.3 | 359.5 | -27.9 | 422.2 | -31.6 | 447.8 | -32.3 |
| MnO/Mn(OH)^+^ | 39.3 | -6.3 | 68.4 | -7.5 | 98.9 | -9.0 | 123.9 | -10.4 | 246.7 | -19.9 | 233.7 | -18.1 | 262.4 | -19.6 | 276.6 | -20.0 |
| MnO/HMnO_2_^-^ | 101.2 | -16.4 | 149.3 | -16.5 | 189.3 | -17.3 | 213.2 | -17.9 | 390.4 | -31.5 | 316.3 | -24.5 | 362.5 | -27.1 | 388.5 | -28.1 |
| MnO/MnO_2_^2-^ | 179.2 | -29.0 | 241.1 | -26.6 | 301.3 | -27.5 | 340.2 | -28.5 | 692.7 | -55.8 | 545.2 | -42.3 | 632.0 | -47.3 | 680.0 | -49.1 |
| CeO_2_/Ce^4+^ | 374.0 | -60.4 | 467.8 | -51.6 | 577.2 | -52.6 | 665.3 | -55.8 | 1100.9 | -88.7 | 879.3 | -68.2 | 1058.0 | -79.2 | 1116.9 | -80.7 |
| CeO_2_/CeOH^3+^ | 287.7 | -46.5 | 362.0 | -40.0 | 444.9 | -40.5 | 512.5 | -43.0 | 857.5 | -69.1 | 724.6 | -56.2 | 845.3 | -63.2 | 889.3 | -64.2 |
| CeO_2_/Ce(OH)_2_^2+^ | 182.3 | -29.5 | 258.8 | -28.6 | 336.2 | -30.6 | 393.5 | -33.0 | 632.8 | -51.0 | 558.6 | -43.3 | 641.9 | -48.0 | 677.6 | -48.9 |
| ^a^ – corresponds to reaction equations in Table S2  ^b^ – unit kJ mol^-1^ | | | | | | | | | | | | | | | | |

Table S7 – Calculated solubility of ZnO, Cu_2_O, Cu, Fe_3_O_4_, MnO, and CeO_2_ in neutral water at 8 different states of water (T, P) along the heating-up process from liquid (50 °C, 35 bar) to supercritical (450 °C, 300 bar)

|  | Solubility (mol kg^-1^) | | | | | | | |
| --- | --- | --- | --- | --- | --- | --- | --- | --- |
|  | 50 °C, 35 bar | 200 °C, 51 bar | 300 °C, 94 bar | 350 °C, 167 bar | 375 °C, 217 bar | 400 °C, 244 bar | 425 °C, 267 bar | 450 °C, 300 bar |
| ZnO | 7.7 × 10^-6^ | 3.9 × 10^-5^ | 1.3 × 10^-4^ | 7.6 × 10^-6^ | 2.3 × 10^-10^ | 6.0 × 10^-10^ | 2.3 × 10^-10^ | 2.0 × 10^-10^ |
| Cu_2_O | 6.3 × 10^-8^ | 1.1 × 10^-5^ | 2.3 × 10^-5^ | 1.1 × 10^-5^ | 3.1 × 10^-10^ | 1.5 × 10^-9^ | 5.9 × 10^-10^ | 6.0 × 10^-10^ |
| Cu | 5.7 × 10^-5^ | 1.8 × 10^-4^ | 5.4 × 10^-6^ | 1.3 × 10^-6^ | 1.0 × 10^-11^ | 2.0 × 10^-10^ | 7.8 × 10^-11^ | 7.4 × 10^-11^ |
| Fe_3_O_4_ | 9.6 × 10^-13^ | 8.2 × 10^-12^ | 8.6 × 10^-12^ | 1.0 × 10^-11^ | 1.4 × 10^-21^ | 2.9 × 10^-15^ | 3.4 × 10^-17^ | 5.1 × 10^-18^ |
| MnO | 4.5 × 10^-4^ | 1.7 × 10^-4^ | 3.0 × 10^-5^ | 5.2 × 10^-6^ | 4.3 × 10^-11^ | 6.7 × 10^-10^ | 1.2 × 10^-10^ | 6.2 × 10^-11^ |
| CeO_2_ | 6.3 × 10^-17^ | 4.9 × 10^-18^ | 5.6 × 10^-20^ | 2.0 × 10^-21^ | 1.3 × 10^-32^ | 3.3 × 10^-24^ | 1.2 × 10^-28^ | 1.0 × 10^-29^ |
|  | | | | | | | | |

# Reference

[1] E.L. Shock, H.C. Helgeson, Calculation of the thermodynamic and transport properties of aqueous species at high pressures and temperatures: Correlation algorithms for ionic species and equation of state predictions to 5 kb and 1000 C, Geochimica et Cosmochimica Acta, 52 (1988) 2009-2036.

[2] J.N. Jocz, L.T. Thompson, P.E. Savage, Catalyst oxidation and dissolution in supercritical water, Chemistry of Materials, 30 (2018) 1218-1229.

[3] Thermophysical Properties of Fluid Systems, in, National Institute of Standards and Technology (NIST), <https://webbook.nist.gov/chemistry/fluid/>.

[4] I. Barin, G. Platzki, Thermochemical data of pure substances, Wiley Online Library, 1989.

[5] W. Wagner, A. Pruß, The IAPWS formulation 1995 for the thermodynamic properties of ordinary water substance for general and scientific use, Journal of Physical and Chemical Reference Data, 31 (2002) 387-535.

[6] E.L. Shock, D.C. Sassani, M. Willis, D.A. Sverjensky, Inorganic species in geologic fluids: correlations among standard molal thermodynamic properties of aqueous ions and hydroxide complexes, Geochimica et Cosmochimica Acta, 61 (1997) 907-950.

[7] D. Fernandez, A. Goodwin, E.W. Lemmon, J. Levelt Sengers, R. Williams, A formulation for the static permittivity of water and steam at temperatures from 238 K to 873 K at pressures up to 1200 MPa, including derivatives and Debye–Hückel coefficients, Journal of Physical and Chemical Reference Data, 26 (1997) 1125-1166.

[8] E.L. Shock, E.H. Oelkers, J.W. Johnson, D.A. Sverjensky, H.C. Helgeson, Calculation of the thermodynamic properties of aqueous species at high pressures and temperatures. Effective electrostatic radii, dissociation constants and standard partial molal properties to 1000 °C and 5 kbar, Journal of the Chemical Society, Faraday Transactions, 88 (1992) 803-826.

[9] E.L. Shock, H.C. Helgeson, D.A. Sverjensky, Calculation of the thermodynamic and transport properties of aqueous species at high pressures and temperatures: Standard partial molal properties of inorganic neutral species, Geochimica et Cosmochimica Acta, 53 (1989) 2157-2183.

[10] G.M. Anderson, D.A. Crerar, Thermodynamics in geochemistry: the equilibrium model, Oxford University Press on Demand, 1993.

[11] W.L. Marshall, E. Franck, Ion product of water substance, 0–1000 C, 1–10000 bars New International Formulation and its background, Journal of Physical and Chemical Reference Data, 10 (1981) 295-304.

1. E-Mail: david.baudouin@psi.ch [↑](#footnote-ref-1)
